# A unique melanocortin-4-receptor signaling profile for obesity-associated constitutively active variants

**DOI:** 10.1530/JME-23-0008

**Published:** 2023-06-12

**Authors:** Rikus Botha, Shree S Kumar, Natasha L Grimsey, Kathleen G Mountjoy

**Affiliations:** 1 Department of Physiology, Faculty of Medical and Health Sciences, University of Auckland, Private Bag, Auckland, New Zealand; 2 Department of Pharmacology, Faculty of Medical and Health Sciences, University of Auckland, Private Bag, Auckland, New Zealand; 3 Centre for Brain Research, Faculty of Medical and Health Sciences, University of Auckland, Private Bag, Auckland, New Zealand; 4 Maurice Wilkins Centre for Biodiscovery, Faculty of Medical and Health Sciences, University of Auckland, Private Bag, Auckland, New Zealand; 5 Department of Molecular Medicine and Pathology, Faculty of Medical and Health Sciences, University of Auckland, Private Bag, Auckland, New Zealand

**Keywords:** GPCR signaling profile, constitutive GPCR, intracellular GPCR signaling, melanocortin-4 receptor, constitutive activity, obesity

## Abstract

The melanocortin-4 receptor (MC4R) plays a critical role in regulating energy homeostasis. Studies on obesogenic human MC4R (hMC4R) variants have not yet revealed how hMC4R maintains body weight. Here, we identified a signaling profile for obesogenic constitutively active H76R and L250Q hMC4R variants transfected in HEK293 cells that included constitutive activity for adenylyl cyclase (AC), cyclic adenosine monophosphate (cAMP) response element (CRE)-driven transcription, and calcium mobilization but not phosphorylated extracellular signal-regulated kinase 1/2 (pERK1/2) activity. Importantly, the signaling profile included impaired α-melanocyte-stimulating hormone-induced CRE-driven transcription but not impaired α-melanocyte-stimulating hormone-induced AC, calcium, or pERK1/2. This profile was not observed for transfected H158R, a constitutively active hMC4R variant associated with overweight but not obesity. We concluded that there is potential for α-melanocyte-stimulating hormone-induced CRE-driven transcription in HEK293 cells transfected with obesogenic hMC4R variants to be the key predictive tool for determining whether they exhibit loss of function. Furthermore, *in vivo*, α-melanocyte-stimulating hormone-induced hMC4R CRE-driven transcription may be key for maintaining body weight.

## Introduction

Obesity is a disease that is in urgent need of treatment. While the G protein-coupled receptor (GPCR), melanocortin-4 receptor (MC4R), is known to play a critical role in regulating energy homeostasis (Stutzmann *et al.* 2008, Wade *et al.* 2021), no safe anti-obesity drugs currently target MC4R (Tao 2009, Montero-Melendez *et al.* 2022). More than 200 distinct human obesity-associated variants are retained intracellularly, and no *in vitro* loss of function (LoF) (measured as Gαs/cyclic adenosine monophosphate (cAMP) accumulation) has been found for many of these variants (Stutzmann *et al.* 2008, Tao 2009, 2010, Wade *et al.* 2021). Recent studies aimed to improve the *in vitro* demonstration of MC4R variant LoF associated with obesity have investigated the efficiency of β-arrestin recruitment to MC4R compared with Gαs/cAMP accumulation or cyclic AMP response element (CRE)-reporter gene activation. One study (Lotta *et al.* 2019) found that the maximum efficacy of β-arrestin recruitment to MC4R, rather than Gαs/cAMP, better explained the variance in the association of MC4R variants with obesity; another study (Wade *et al.* 2021) did not. Gillyard *et al.* showed that the β-arrestin recruitment assay did not measure a physiological response (Gillyard *et al.* 2019). Therefore, after two decades, no study has elucidated how the human MC4R (hMC4R) signals to regulate body weight. A new approach is needed.

Surprisingly, six hMC4R variants with constitutive activity (i.e. receptor activation in the absence of ligand; basal activity relative to wild-type (WT)) have been associated with obesity (Tao 2009, 2014, Tao* et al.* 2010, Mo & Tao 2013). These obesogenic variants pose a conundrum since enhanced hMC4R signaling was predicted to associate with a lean phenotype. Two of these variants, H76R (Gillyard *et al.* 2019) and L250Q (Xiang *et al.* 2006), were shown to have reduced agonist-activated CRE-driven transcription activity *in vitro*.

Therefore, we hypothesized that a constitutively active hMC4R LoF conformational state exists with a signaling profile distinct from WT hMC4R and non-constitutively active hMC4R LoF variants, and this would cause obesity. Here, we compared basal and α-melanocyte-stimulating hormone (α-MSH)-activated signaling between WT hMC4R and published hMC4R constitutively active variants associated with obesity, non-constitutively active hMC4R variants associated with obesity, constitutively active hMC4R variants not associated with obesity, and non-constitutively active hMC4R variants associated with reduced risk of developing obesity (Supplementary Fig. 1 and Supplementary Table 1, see section on supplementary materials given at the end of this article). We included WT hMC4R co-expressed with human melanocortin-2 receptor accessory protein alpha (hMRAPα) or human melanocortin-2 receptor accessory protein 2 (hMRAP2) since we, and others, previously identified hMC4R co-expressed with hMRAPα exhibited increased constitutive adenylyl cyclase (AC) activity *in vitro* (Kay *et al.* 2013*a*, 2015, Ji & Tao 2022). In contrast, hMRAP2 (Kay *et al.* 2015, Schonnop *et al.* 2016) had no significant effect on basal MC4R coupling to cAMP or CRE-reporter activity when accessory protein and receptor were co-expressed *in vitro* in a 1:1 DNA ratio. Therefore, if we could show hMRAPα-induced constitutively active hMC4R shared the same signaling profile as obesogenic constitutively active variants, this would suggest that the development of anti-obesity therapeutics aimed at increasing WT hMC4R basal activity would likely fail to decrease body weight.

Our data confirmed constitutive activity for both AC and CRE-reporter transcription pathways for two of the six previously reported obesogenic hMC4R variants and one hMC4R variant not associated with obesity. Importantly, the data support our hypothesis that obesogenic constitutively active hMC4R variants exhibit an LoF signaling profile distinct from WT hMC4R, non-constitutively active hMC4R LoF variants, and non-obesogenic variants.

## Materials and methods

### Experimental model

Other studies characterized the expression and signaling for the hMC4R variants we selected to study (Supplementary Table 1). Here, we developed HA (hemagglutinin) epitope-tagged hMC4R variants to validate published results. We included D90N as a negative control due to its known failure to activate Gαs signaling (Buch *et al.* 2009) and hMRAP2, or no MRAP co-expression, as a negative control for hMRAPα-induced constitutive activity (Kay *et al.* 2015).

### Construction of HA-hMC4R variants, hMRAPα, and hMRAP2

Point mutations to create hMC4R variants were introduced into HA-hMC4R-WT using overlap extension PCR (Vallejo *et al.* 2008) as described in Supplementary methods and Supplementary Table 2. hMRAPα and hMRAP2 cloned into pcDNA3.1 were developed previously (Kay *et al.* 2013*a*).

### Cell culture

Culturing HEK293 and GT1–7 cell lines used is described in Supplementary methods.

### Biochemical reagents

Biochemical reagents used are listed in Supplementary Table 3.

### CRE-β-galactosidase (β-Gal) reporter gene assay

CRE-reporter gene transcription activity was studied as an indirect measure of hMC4R coupling to Gαs since the CRE-reporter gene is activated by Gαs, Gαi, Gαq, and β-arrestin (Chen *et al.* 1995, Fitzgerald *et al.* 1999, Manson *et al.* 2011). The method is described in Supplementary methods.

### AC assay

AC activity was studied as a direct measure of hMC4R coupling to Gαs activation and was measured as previously described (Mountjoy *et al.* 1999, Kay *et al.* 2013*a*) and in Supplementary methods.

### Pre-treatment of cells with pertussis toxin (PTX)

To determine whether hMC4R variant constitutive activity results from dysfunctional Gαi, cells were pre-treated with PTX to inhibit Gαi, as described in Supplementary methods.

### Calcium assay

hMC4R coupling to mobilization of intracellular calcium ([Ca^2+^]_i_) was measured using Fura 2AM in HEK293 cells stably expressing WT hMC4R or variant of interest, as previously described (Kumar *et al.* 2021). To study hMRAPα-induced hMC4R constitutive activity, either pcDNA3.1 (control) or hMRAPα was transiently expressed in HEK293 cells stably expressing WT hMC4R, as described in Supplementary methods. We monitored baseline (no injection), vehicle, and α-MSH (10^−10^ M to 10^−6^ M)-stimulated fluorescence at 340 and 380 for 30 s before injections of vehicle or α-MSH injections and then for 50 s post-injection. An example of data collected is shown in Supplementary Fig. 3.

### Pre-treatment of cells with EPAC1/2 inhibitor ESI-09

Exchange factors directly activated by cAMP1/2 (known as EPAC1/2) mediate [Ca^2+^]_i_ mobilization and MC4R-induced CRE-gene activity (Dzhura *et al.* 2010, Glas *et al.* 2016). To determine whether EPAC1/2 mediated hMC4R-driven [Ca^+2^]_i_ mobilization and CRE-β-Gal reporter gene activity, we pre-treated cells with ESI-09, an EPAC1/2 inhibitor, as described in Supplementary methods.

### Lysate generation and western blots

Cell lysates and western blots for extracellular signal-regulated kinase 1/2 (ERK1/2), phosphorylated ERK1/2 (pERK1/2,) and total cellular HA-hMC4R protein expression were prepared using methods modified from a previous study (Kay *et al.* 2013*b*) and fully described in Supplementary methods.

### Cell-surface HA-hMC4R protein expression

Cell-surface HA-hMC4R protein expression was measured using mouse anti-HA.11 monoclonal antibody and ELISA, as described in Supplementary methods.

### Pre-treatment of cells with Dyngo4a

Dyngo4a inhibits clathrin-mediated endocytosis (Villasenor *et al.* 2016). To determine whether hMC4R variant constitutive activity arises from either cell-surface or intracellular locations, we pre-treated cells with Dyngo4a, as described in Supplementary methods.

### Statistical analysis

GraphPad Prism 7.0 software was used to generate graphs and perform statistical analyses described in Supplementary methods.

## Results

### Validation of constitutive CRE-β-Gal reporter gene activity for hMC4R variants, H76R, L250Q and H158R, and WT hMC4R co-expressed with hMRAPα

To validate published hMC4R constitutive activity for six obesity-associated hMC4R variants (H76R, S127L, P230L, L250Q, F280L, and S295P) and two non-obesity-associated variants (D146N and H158R) (Supplementary Table 1 and Supplementary Fig. 1), we performed a screen of basal CRE-β-Gal reporter gene activity in HEK293 cells. We compared the above hMC4R variants with (i) WT hMC4R, (ii) two reported non-constitutively active hMC4R variants associated with reduced risk of developing obesity (V103I and I251L), (iii) five hMC4R reported non-constitutively active obesity-associated variants (R7H, R18L, T150I, A154D, and R305S), and (iv) hMC4R variant, D90N (see Supplementary Table 1).

**Figure 1:**
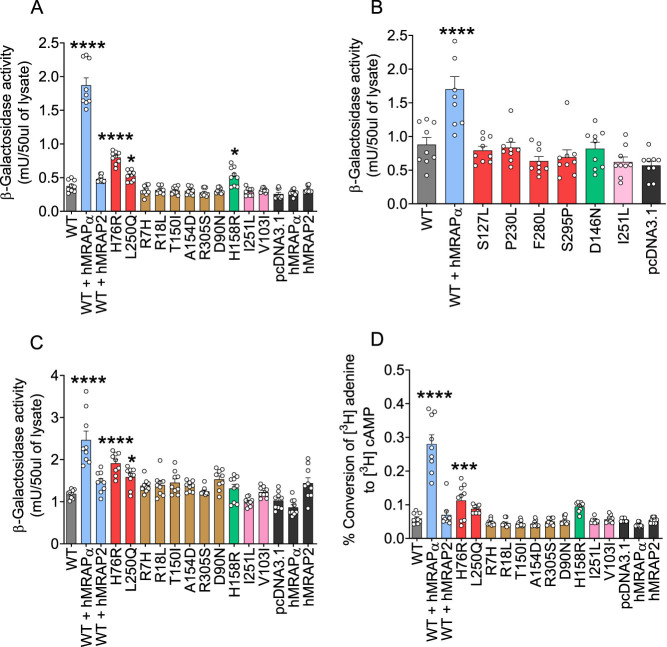
Validation of CRE-β-Gal reporter gene and AC constitutive activity for WT hMC4R, hMC4R variants, and WT hMC4R co-expressed with hMRAPα transiently expressed in HEK293 or GT1–7 cells. Basal CRE-β-Gal reporter gene activity measured for WT hMC4R ± hMRAPα or hMRAP2 and hMC4R variants transiently transfected in HEK293 cells (A, B) and GT1–7 cells (C). The CRE-β-Gal reporter gene assays were performed with triple transfections using DNAs for CRE-β-Gal reporter gene, hMC4R, and pcDNA3.1, hMRAPα, or hMRAP2. AC activity measured for WT hMC4R + hMRAPα or hMRAP2 and hMC4R variants + pcDNA3.1 transiently transfected in HEK293 cells (D). The AC assays were performed with double transfections using DNAs for WT hMC4R and pcDNA3.1, hMRAPα, or hMRAP2. pcDNA3.1, hMRAPα, and hMRAP2 were each transfected alone for all assays to serve as negative controls. Data were pooled from triplicate wells of three independent experiments and shown as mean ± s.e.m. We performed one-way ANOVA and Dunnett's posthoc analysis to determine significant differences compared to WT hMC4R. ^*^, *P* < 0.05; ^***^, *P* < 0.001; ^****^, *P* < 0.0001.

Our data confirmed constitutive CRE-β-Gal reporter gene activity for hMC4R variants H76R (increased by 113%; *P* < 0.0001), L250Q (increased by 41%; *P* = 0.0177), and H158R (increased by 38%; *P* = 0.0338) compared to WT hMC4R (Fig. 1A). We did not confirm constitutive CRE-β-Gal reporter gene activity for any other hMC4R variant reported to exhibit constitutive activity (S127L, D146N, P230L, F280L, and S295P). Paisdzior *et al.* also failed to confirm constitutive activity for S127L (Paisdzior *et al.* 2020). We did not show constitutive activity for any hMC4R variant (R7H, R18L, V103I, T150I, A154D, I251L, and R305S) reported not to exhibit constitutive activity (Fig. 1A and B). Our data showed for the first time that WT hMC4R co-expressed with hMRAPα, but not hMRAP2, exhibited constitutive CRE-β-Gal reporter activity (increased by 398%; *P* < 0.0001) compared to WT hMC4R expressed alone (Fig. 1A).

We also performed CRE-β-Gal reporter gene assays following transient transfection into mouse hypothalamic GT1–7 cells (Mellon *et al.* 1990). Our data confirmed constitutive CRE-β-Gal reporter gene activity for hMC4R variants, H76R (increased by 61.0%; *P* < 0.0001) and L250Q (increased by 38.8%; *P* < 0.05), but not H158R (increased by 10.7%; *P* = 0.9837), compared to WT hMC4R (Fig. 1C). Therefore, H158R constitutive activity appears cell-type dependent. Similar to responses observed in HEK293 cells, hMRAPα but not hMRAP2 co-expressed with WT hMC4R induced WT hMC4R constitutive CRE-β-Gal reporter gene activity in GT1–7 cells (increased by 107.7%; *P* < 0.0001) (Fig. 1C).

### Validation of constitutive AC activity for hMC4R variant, H76R, and WT hMC4R co-expressed with hMRAPα

We next determined whether constitutive activity was observed for AC activity, which is upstream of CRE-reporter gene activation. Using HEK293 cells, we showed constitutive AC activity for H76R compared to WT hMC4R (increased by 88.7%; *P* < 0.001) (Fig. 1D). H158R and L250Q variants showed clear trends for constitutive AC activity (L250Q: increased by 47.6%; *P* = 0.2031; H158R: increased by 59.1%; *P* = 0.0543) (Fig. 1D). We did not detect constitutive AC activity in HEK293 cells for any other hMC4R variant (Fig. 1D). Furthermore, we confirmed that hMRAPα, but not hMRAP2, co-expressed with WT HA-hMC4R induced WT hMC4R constitutive AC activity (increased by 371.1%; *P* < 0.0001) (Kay *et al.* 2015) (Fig. 1D). Ji *et al.* (Ji & Tao 2022) also showed that hMRAPα co-expressed with hMC4R in HEK293T cells induced constitutive activity, but they showed three hMRAP2 isoforms each co-transfected with hMC4R, significantly decreased basal intracellular cAMP levels. Their different MRAP2 response may be due to Ji *et al.* (Ji & Tao 2022) transfecting MC4R/MRAP plasmids in a 1:5 ratio vs the 1:1 ratio used here.

Overall, our data indicate that there are only three naturally occurring hMC4R variants (H76R, L250Q, and H158R) that exhibit constitutive activity for Gαs-cAMP and/or CRE-driven transcription signaling compared to WT hMC4R.

### α-MSH induced AC but not CRE-β-Gal reporter gene activity for H76R, L250Q, and WT hMC4R co-expressed with hMRAPα but induced both AC and CRE-β-Gal reporter gene activity for WT hMC4R and H158R variant

**Figure 2:**
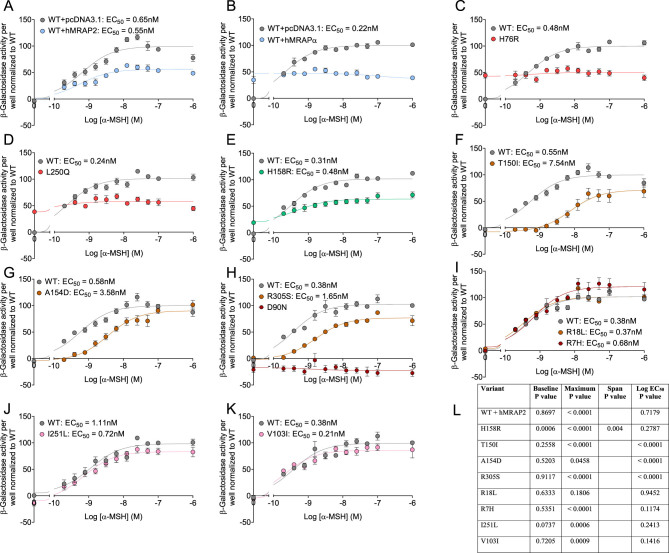
Functional coupling of α-MSH-stimulated CRE-β-Gal reporter gene activity for WT hMC4R, hMC4R variants, and WT hMC4R co-expressed with hMRAPα transiently expressed in HEK293 cells. The CRE-β-Gal reporter gene assays were performed with triple transfections using DNAs for CRE-β-Gal reporter gene, WT hMC4R + pcDNA3.1, hMRAPα, or hMRAP2. We measured CRE-β-Gal reporter gene activity for WT hMC4R + hMRAP2 (A), hMRAPα (B), and hMC4R variants + pcDNA3.1 transiently transfected in HEK293 cells (B-K). We pooled data from three or four independent α-MSH concentration–response curves showing stimulation of β-Gal activity and normalized fitted curves to the top and bottom of WT hMC4R curves using GraphPad Prism. Summary of significance for comparative CRE-β-Gal reporter gene activity best-fit curve fitting for WT hMC4R vs hMC4R variants and WT hMC4R co-expressed with hMRAP2 (L). Data for H76R, L250Q, and WT hMC4R co-expressed with hMRAPα did not generate best-fit curves, and therefore no data for these are included in the summary of significance. Data are shown as mean ± s.e.m. The non-parametric sum of squares *f*-test was used to determine significance for parameters derived from curve fitting. The span parameter was derived from the three to four independent curves, and a paired Student’s *t*-test was used to determine significant differences.

α-MSH induced a classic sigmoidal concentration–response curve activating CRE-β-Gal reporter gene activity for WT hMC4R, with or without co-expression of hMRAP2 (Fig. 2A). In contrast, α-MSH did not induce CRE-β-Gal reporter gene sigmoidal concentration–response curves for hMC4R co-expressed with hMRAPα (Fig. 2B), H76R (Fig. 2C) or L250Q (Fig. 2D). However, the non-obesogenic constitutively active hMC4R variant, H158R, exhibited α-MSH-induced sigmoidal concentration–response curve together with constitutive activity (Fig. 2E and L). While previous studies showed sigmoidal concentration curves for α-MSH-inducing CRE-driven reporter gene for H76R (Gillyard *et al.* 2019) or L250Q (Vaisse *et al.* 2000, Proneth *et al.* 2006, Xiang *et al.* 2006), these responses were significantly impaired compared with WT hMC4R. The reason they observed sigmoidal concentration curves and we did not is likely due to the use of a different CRE-driven reporter gene from that used in this study. Non-constitutively active hMC4R variants showed sigmoidal α-MSH concentration-dependent response curves for CRE-β-Gal reporter gene activity with varying potencies and efficacies (Fig. 2F, G, H, I, J, K and L). As expected, α-MSH did not stimulate CRE-β-Gal reporter gene activity for control hMC4R variant, D90N (Buch *et al.* 2009) (Fig. 2H).

The maximum response was decreased relative to WT hMC4R for H158R, T150I, R305S, V103I, I251L, and WT hMC4R+hMRAP2 and increased for R7H (Fig. 2L). The baseline response was significantly increased relative to WT hMC4R for H158R, and the span for H158R α-MSH-induced sigmoidal curve compared to WT hMC4R was significantly different (Fig. 2L). Therefore, the maximum α-MSH-induced concentration response for H158R was reduced despite H158R constitutive CRE-β-Gal reporter gene activity (Fig. 2L), a response that was not observed for H76R and L250Q.

**Figure 3:**
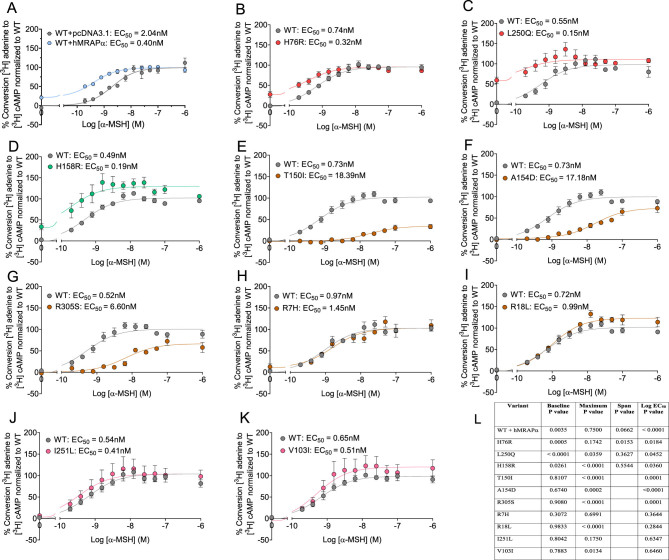
Functional coupling of α-MSH stimulated AC activity for WT hMC4R, hMC4R variants, and WT hMC4R co-expressed with hMRAPα transiently expressed in HEK293 cells. The AC assays were performed with double transfections using DNAs for WT hMC4R + pcDNA3.1 or hMRAPα. We measured AC activity for WT hMC4R + pcDNA3.1 or hMRAPα (A) and hMC4R variants + pcDNA3.1 (B-J) transiently transfected in HEK293 cells. We pooled data from three or four independent α-MSH concentration–response curves showing stimulation of AC activity and normalized fitted curves to the top and bottom of WT hMC4R curves using GraphPad Prism. Summary of significance for comparative AC activity best-fit curve fitting for WT hMC4R vs hMC4R variants and WT hMC4R co-expressed with hMRAPα (L). Data are shown as mean ± s.e.m. The non-parametric sum of squares *f*-test was used to determine significance for parameters derived from curve fitting. The span parameter was derived from the three to four independent curves, and pairwise Student’s *t*-test was used to determine significant differences.

In contrast to CRE-β-Gal reporter gene activity, α-MSH induced concentration-dependent sigmoidal response curves for AC activity for WT hMC4R co-expressed with hMRAPα, H76R, and L250Q (Fig. 3A-C) (Kay *et al.* 2013*a*, 2015). The constitutively active hMC4R variant, H158R, also exhibited an α-MSH-induced sigmoidal AC response curve (Fig. 3D). Non-constitutively active hMC4R variants all exhibited sigmoidal α-MSH concentration-dependent response curves for AC activity with varying potencies and efficacies (Fig. 3E, F, G, H, I, J, K and L). The maximum AC response was significantly increased for H158R compared to WT hMC4R (Fig. 3D and L). The baseline AC response was significantly increased relative to WT hMC4R (constitutive activity) for H76R, L250Q, H158R, and WT hMC4R co-expressed with hMRAPα (Fig. 3L). The span for H158R α-MSH-induced sigmoidal curve compared to WT hMC4R was not significantly different (Fig. 3L), indicating H158R constitutive AC activity increased the α-MSH-induced maximum AC response (Fig. 3L).

Importantly, we showed impaired α-MSH signaling for obesogenic constitutively active hMC4R variants. Similar to WT hMC4R, both H76R and L250Q responded to α-MSH concentration-dependent-induced AC activity. However, unlike WT hMC4R, neither H76R nor L250Q responded to α-MSH concentration-dependent induced CRE-β-Gal reporter gene activity. The non-obesogenic constitutively active H158R variant responded to both α-MSH-induced AC and CRE-β-Gal reporter gene activity. We summarized these results in Supplementary Table 4 and Supplementary Table 5. We next performed studies to understand mechanisms causing constitutive activity together with impaired α-MSH-induced CRE-β-Gal reporter gene activity.

### Neither constitutive activity nor impaired α-MSH-induced CRE-β-Gal reporter gene activity for obesogenic constitutively active hMC4R variants arises due to dysfunctional Gαi coupling

hMC4R is known to functionally couple to Gαi and Gαs in HEK293 and GT1–7 cells, and the presence of PTX is known to enhance α-MSH-stimulated cAMP production (Buch *et al.* 2009). We showed PTX inhibition of Gαi significantly enhanced α-MSH-induced, but not basal, CRE-β-Gal gene reporter activity for WT hMC4R (Supplementary Fig. 2A and D). However, PTX significantly increased basal (constitutive) CRE-β-Gal gene reporter activity for H76R and WT hMC4R co-expressed with hMRAPα (Supplementary Fig. 2B, C and D). Therefore, we concluded that neither constitutive activity nor impaired α-MSH-induced CRE-β-Gal reporter gene activity arises due to dysfunctional Gαi coupling.

### Disrupted [Ca^2+^]_i_ signaling does not appear to contribute to impaired α-MSH-induced CRE-β-Gal reporter gene activity

**Figure 4:**
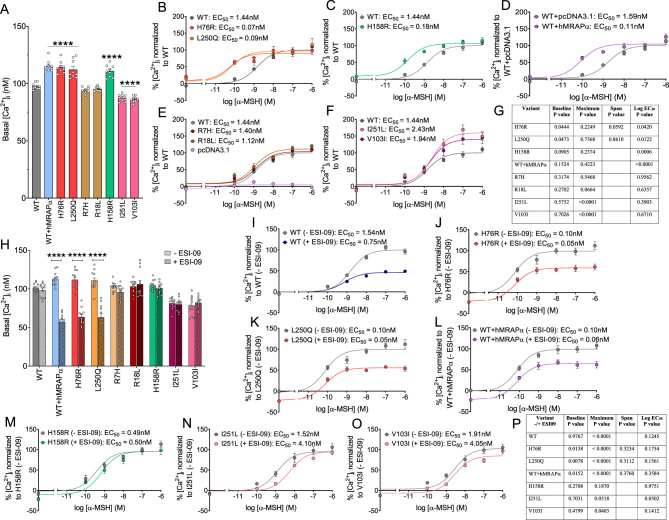
Functional coupling of α-MSH-induced mobilization of [Ca^2+^]_i_ for WT hMC4R, hMC4R variants, and WT hMC4R co-expressed with hMRAPα expressed in HEK293 cells. We performed Fura-2 AM calcium assays in stable cell lines expressing DNAs for WT hMC4R or hMC4R variants and the stably expressing hMC4R cell line transiently transfected with DNAs for pcDNA3.1 or hMRAPα. We measured basal (A) and α-MSH-induced (B-F) [Ca^2+^]_i_ for WT hMC4R, hMC4R variants, and WT hMC4R co-expressed with hMRAPα. The statistical significance for basal [Ca^2+^]_i_ in panel A was determined using one-way ANOVA and compared to WT hMC4R. ^****^ *P* < 0.0001. We pooled data from three independent α-MSH concentration–response curves showing stimulation of [Ca^2+^]_i_ and normalized fitted curves to the top and bottom of WT hMC4R curves using GraphPad Prism. The raw Fura-2 AM fluorescence data and dynamic [Ca^2+^]i responses are shown in Supplementary Figure 3 for one of the three independent α-MSH concentration–response experiments performed on WT hMC4R. Summary of significance for comparative [Ca^2+^]_i_ best-fit curve fitting for WT hMC4R vs hMC4R variants and WT hMC4R co-expressed with hMRAPα (G). Data are shown as mean ± s.e.m. The non-parametric sum of squares *f*-test was used to determine significance for parameters derived from curve fitting. The span parameter was derived from the three independent curves, and a paired Student’s *t*-test was used to determine significant differences. We measured basal (H) and α-MSH-induced [Ca^2+^]_i_ for WT hMC4R (I), H76R (J), L250Q (K), WT hMC4R co-expressed with hMRAPα (L), H158R (M), I251L (N), and V103I (O) following treatment with 10 µM ESI-09 or vehicle. The statistical significance for basal [Ca^2+^]_i_ in panel H was determined using Student's *t*-test comparing vehicle with ESI-09 treatment. ^****^P < 0.0001. We pooled data from three independent α-MSH concentration–response curves showing stimulation of [Ca^2+^]_i_ and normalized fitted curves to the top and bottom of WT hMC4R curves using GraphPad Prism. Summary of significance for comparative [Ca^2+^]_i_ best-fit curve fitting for vehicle vs ESI-09 treatments (P). Data are shown as mean ± s.e.m. The non-parametric sum of squares *f*-test was used to determine significance for parameters derived from curve fitting. The span parameter was derived from the three independent curves, and pairwise Student’s *t*-test was used to determine significant differences.

We next investigated a potential role for MC4R regulation of [Ca^2+^]i, causing the signaling mismatch between α-MSH-stimulated AC and CRE-β-Gal reporter gene activity. An increase in [Ca^2+^]_i_ is critical for CRE-driven gene transcription (Ginty 1997), and previously we showed α-MSH-induced MC4R transfected in HEK293 cells to increase [Ca^2+^]_i_ partially through Gαs signaling (Mountjoy *et al.* 2001, Kumar *et al.* 2021). Here, we discovered constitutive [Ca^2+^]_i_ for obesogenic constitutively active H76R (increased by 17.1%; *P* < 0.0001) and L250Q (increased by 14.9% ; *P* < 0.0001) and non-obesogenic constitutively active H158R (increased by 13.5%; *P* < 0.0001) compared to WT hMC4R (Fig. 4A). WT hMC4R co-expressed with hMRAPα also showed constitutive [Ca^2+^]_i_ (increased by 13.3%; *P* < 0.0001) compared to WT hMC4R alone (Fig. 4A). Basal [Ca^2+^]_i_ for non-constitutively active hMC4R LoF variants, R7H and R18L, was no different to WT hMC4R (Fig. 4A). Therefore, constitutive Ca^2+^ signaling coincides with the presence of constitutive AC and CRE-β-Gal reporter gene activities.

We showed α-MSH-induced [Ca^2+^]_i_ more potently (log EC_50_ significantly decreased) compared with WT hMC4R for H76R, L250Q, H158R, and WT hMC4R co-expressed with hMRAPα (Fig. 4B, C and D) but not for R7H, R18L, I251L, and V103I (Fig. 4E, F and G). This was similar to the enhanced potency of α-MSH activating AC activity for these variants (Fig. 3A, B, C and D). The baseline [Ca^2+^]_i_ response was significantly increased relative to WT hMC4R for H76R and L250Q (Fig. 4B and G), but the span was not significantly different for H76R and L250Q compared with WT hMC4R (Fig. 4G). The baseline [Ca^2+^]_i_ response appeared to be increased for H158R and WT hMC4R co-expressed with hMRAPα compared with WT hMC4R, but these were not significant differences (Fig. 4C, D and G). These data, summarized in Supplementary Table 6, support the fact that α-MSH-induced MC4R regulation of [Ca^2+^]_i_ is partially dependent on Gαs/AC signaling. However, we cannot rule out that impaired Ca^2+^ signaling independent of Gαs/AC or CRE-driven transcription signaling impairs α-MSH-induced CRE-β-Gal reporter gene transcription.

Surprisingly, our data showed significantly reduced basal [Ca^2+^]_i_ for V103I (decreased by 11.5%; *P* < 0.0001) and I251L (decreased by 10.3%; *P* < 0.0001) compared to WT hMC4R (Fig. 4A) and a trend for reduced basal [Ca^2+^]_i_ for V103I and I251L compared to WT hMC4R in the α-MSH-induced [Ca^2+^]_i_ experiments (Fig. 4F). The α-MSH-induced maximum [Ca^2+^]_i_ response for V103I and I251L was significantly increased relative to WT hMC4R (Fig. 4F and G). These findings were unexpected based on our data suggesting a link between Gαs and [Ca^2+^]_i_ for constitutively active hMC4R variants. Neither V103I nor I251L compared to WT hMC4R showed reduced basal AC or CRE-β-Gal reporter gene activity. While α-MSH significantly increased maximum AC activity for V103I (Fig. 3K and L) but not I251L (Fig. 3J and L), α-MSH significantly decreased maximum CRE-β-Gal reporter gene activity for both V103I (Fig. 2K and L) and I251L (Fig. 2J and L) compared to WT hMC4R. Future studies are required to investigate how V103I and I251L variants regulate [Ca^2+^]_i_ in HEK293 cells and whether this regulation contributes to reduced risk of developing obesity. Here, our focus was to understand obesogenic constitutively active hMC4R variant [Ca^2+^]_i_ regulation and impairment of α-MSH-induced CRE-driven transcription.

### EPAC1/2 signaling partially mediated WT hMC4R and obesogenic constitutively active hMC4R variants induction of [Ca^2+^]_i_

Our data here and previously (Mountjoy *et al.* 2001), suggest WT hMC4R can engage both cAMP/protein kinase A (PKA) and cAMP/EPAC1/2 signaling pathways to induce [Ca^2+^]_i_ in HEK293 cells. ESI-09 inhibition of EPAC1/2 significantly reduced basal [Ca^2+^]_i_ for H76R (decreased by 43.2%; *P* < 0.0001), L250Q (decreased by 42.9%; *P* < 0.0001) and WT hMC4R co-expressed with hMRAPα (decreased by 49.2%; *P* < 0.0001) (Fig. 4H). ESI-09 significantly reduced α-MSH-induced maximum [Ca^2+^]_i_ response (decreased by 43.7%; *P* < 0.0001) but not basal [Ca^2+^]i for WT hMC4R (Fig. 4I). ESI-09 pre-treatment also significantly decreased α-MSH-induced maximum [Ca^2+^]_i_ response for H76R, L250Q, and WT hMC4R co-expressed with hMRAPα (Fig. 4J, K, L and P). However, since the span for the α-MSH-induced [Ca^2+^]_i_ responses were not significantly different following ESI-09 treatment (Fig. 4P), the significantly reduced α-MSH-induced maximum [Ca^2+^]_i_ responses for H76R, L250Q, and WT hMC4R co-expressed with hMRAPα simply reflect significantly reduced basal [Ca^2+^]_i_. ESI-09 pre-treatment did not change basal or maximum α-MSH-induced [Ca^2+^]_i_ responses for the H158R variant (Fig. 4M and P). Overall, our data indicate that α-MSH-induced WT hMC4R and basal obesogenic constitutively active hMC4R variants regulation of [Ca^2+^]_i_ is partially dependent on EPAC1/2.

ESI-09 pre-treatment did not significantly reduce the basal [Ca^2+^]_i_ response for I251L or V103I, but it significantly reduced the α-MSH-induced maximum [Ca^2+^]_i_ response for both variants (Fig. 4N, O, and P). Future work is required to determine whether the pathway independent of EPAC1/2 involves PKA or is independent of Gαs signaling.

### No evidence for impaired EPAC1/2 signaling causing impaired α-MSH-driven transcription

Glas *et al.* showed that EPAC1/2 plays a critical role in MC4R-induced CRE-reporter gene activation (Glas *et al.* 2016). We showed that EPAC1/2 contributes to constitutive CRE-β-Gal reporter gene activity for H76R and WT hMC4R co-expressed with hMRAPα (Supplementary Fig. 4). ESI-09 pre-treatment significantly reduced basal CRE-β-Gal reporter gene activity for H76R (decreased by 30.1%; *P* = 0.0139) and WT hMC4R co-expressed with hMRAPα (decreased by 26.6%; *P* = 0.0463) (Supplementary Fig. 4A). However, ESI-09 did not reduce L250Q or H158R constitutive CRE-β-Gal gene reporter activity (Supplementary Fig. 4A).

We showed that ESI-09 significantly reduced α-MSH-induced WT hMC4R maximum CRE-β-Gal reporter gene activity (decreased by 45.7%; *P* < 0.0001) (Supplementary Fig. 4B). There was no α-MSH-induced CRE-β-Gal reporter gene activity for H76R with or without ESI-09 (Supplementary Fig. 4C and D). Therefore, our data indicate that EPAC1/2 contributes to H76R constitutive CRE-β-Gal reporter gene activity and α-MSH-induced maximum WT hMC4R CRE-β-Gal reporter gene activity. Future studies are required to understand why EPAC1/2 does not contribute to L250Q and H158R constitutive CRE-reporter gene activity. Regardless of this, the cause of failure of α-MSH to induce CRE-β-Gal reporter gene activity for H76R does not appear to result from impaired EPAC1/2 signaling since ESI-09 significantly reduced H76R constitutive CRE-β-Gal reporter gene activity but did not restore α-MSH-induced CRE-β-Gal reporter gene activity.

### No evidence for impaired pERK1/2 signaling causing impaired α-MSH-induced CRE-driven transcription

**Figure 5:**
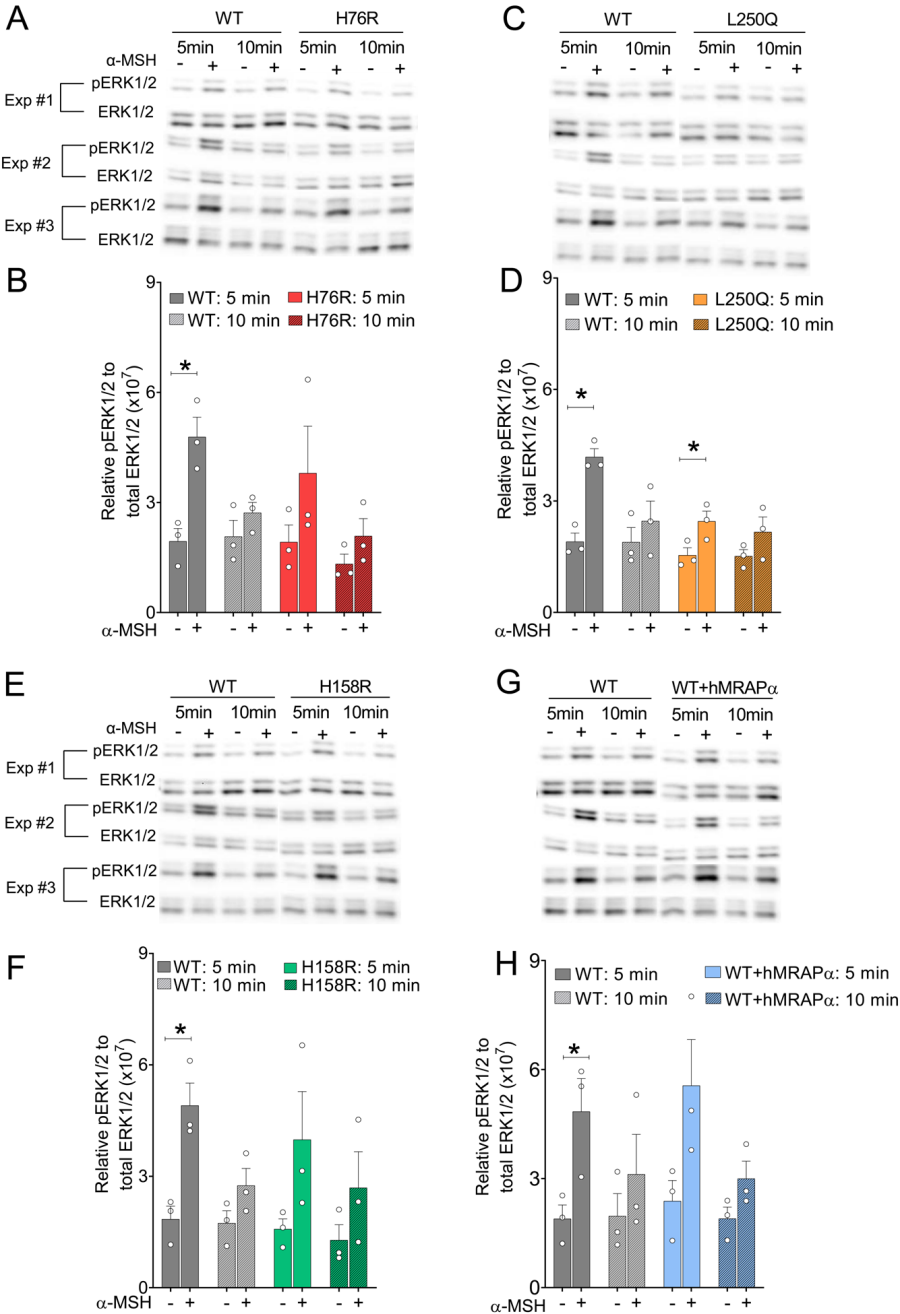
Functional coupling of α-MSH stimulated pERK1/2 for WT hMC4R, hMC4R variants, and WT hMC4R co-expressed with hMRAPα transiently expressed in HEK293 cells. We prepared cell lysates from HEK293 cells following transient double transfections using DNAs for WT hMC4R + pcDNA3.1 (A–H), H76R + pcDNA3.1 (A, B), L250Q + pcDNA3.1 (C, D), H158R + pcDNA3.1 (E, F), and WT hMC4R + hMRAPα (G, H). We performed three independent experiments with one sample for each transfection per experiment. We electrophoretically separated pERK1/2 and ERK1/2 proteins by SDS-PAGE, transferred them to membranes, and then detected them using anti-pERK1/2 and anti-ERK1/2 antibodies, respectively, and western blotting. We show data as mean ± s.e.m. We performed one-way ANOVA and determined significant or near-significant, differences as follows: ANOVA for B, *P* = 0.0749; ANOVA for D, *P* = 0.0008 + Tukey’s multiple comparison test for WT hMC4R without α-MSH vs WT hMC4R with α-MSH for 5 min, *P* = 0.003, and WT hMC4R with α–MSH vs L250Q with α-MSH for 5 min, *P* = 0.0297; ANOVA for F, *P* = 0.0724; ANOVA for H, *P* = 0.0236. The statistical significance shown on the graphs was determined using the non-parametric pairwise Student’s *t*-test between vehicle and α-MSH treatment at either 5 or 10 min. ^*^P < 0.05.

α-MSH is known to stimulate MC4R-induced pERK1/2 (Daniels *et al.* 2003, Vongs *et al.* 2004, Mo & Tao 2013, He & Tao 2014) which is mediated by Gαs signaling (Brouwers *et al.* 2021). Therefore, we asked whether impaired pERK1/2 signaling could explain the impaired α-MSH-induced CRE-β-Gal reporter gene activity for H76R and L250Q hMC4R variants. Our data showed that baseline pERK1/2 levels for H76R (Fig. 5A and B), L250Q (Fig. 5C and D), H158R (Fig. 5E and F), and WT hMC4R co-expressed with hMRAPα (Fig. 5G and H) were no different to WT hMC4R baseline pERK1/2 levels. In this respect, our data differ from a prior study that found six constitutively active (cAMP or CRE-driven transcription) hMC4R mutants to exhibit constitutive pERK1/2 activity compared to WT hMC4R (Mo & Tao 2013). This difference may be due to using 1 h serum starvation of cells here vs Mo *et al.* (Mo & Tao 2013) using overnight serum starvation. We determined that pERK1/2 activity was sensitive to serum starvation (decreased activity) and cell culture media change (increased activity). Therefore, differences in the handling of cells would impact pERK1/2 activity.

As expected, exposure to α-MSH (1μM) for 5, but not 10, minutes, significantly induced pERK1/2 activity for WT hMC4R (*P* = 0.0299) (Fig. 5A, B, C, D, E, F, G and H). Five, but not 10, minutes of exposure to α-MSH (1 μM) also significantly induced pERK1/2 activity for L250Q (*P* = 0.0175) (Fig. 5C and D). Our data did not reach significance but showed trends for 5, but not 10, min exposure to α-MSH (1 μM) to increase pERK1/2 activity for H76R (*P* = 0.0824) (Fig. 5A and B), H158R (*P* = 0.1344) (Fig. 5E and F), and WT hMC4R co-expressed with hMRAPα (*P* = 0.4140) (Fig. 5G and H). Overall, we showed that basal and α-MSH-activated pERK1/2 activity for H76R, L250Q, H158R, and WT hMC4R co-expressed with hMRAPα were similar to data generated for WT hMC4R. Therefore, impaired α-MSH-induced pERK1/2 unlikely contributed to impaired α-MSH-induced CRE-driven transcription.

### Neither reduced cell-surface nor intracellular hMC4R expression appeared to underlie the obesogenic constitutively active hMC4R variants impaired α-MSH-induced CRE-β-Gal reporter gene activity

Gαs signaling occurs at the cell surface and in endosomes (Irannejad & von Zastrow 2014, Sutkeviciute & Vilardaga 2020, Brouwers *et al.* 2021). α-MSH only binds cell-surface MC4R to activate Gαs (Brouwers *et al.* 2021). Importantly, endosomal, but not cell surface, Gαs signaling regulates CRE-driven gene transcription (Tsvetanova & von Zastrow 2014, Jensen *et al.* 2017, Thomsen *et al.* 2018, Canals *et al.* 2019). Based on previous studies (Supplementary Table 1 and (Kay *et al.* 2013*b*)), we predicted that WT hMC4R is expressed at the cell surface in the basal state, whereas H76R, L250Q, H158R, and hMRAPα-induced constitutively active hMC4R are expressed both at the cell surface and intracellularly. Hence, in the basal state, the cell-surface constitutively active receptor could generate cAMP and the intracellular constitutively active receptor could generate CRE-driven gene transcription.

**Figure 6:**
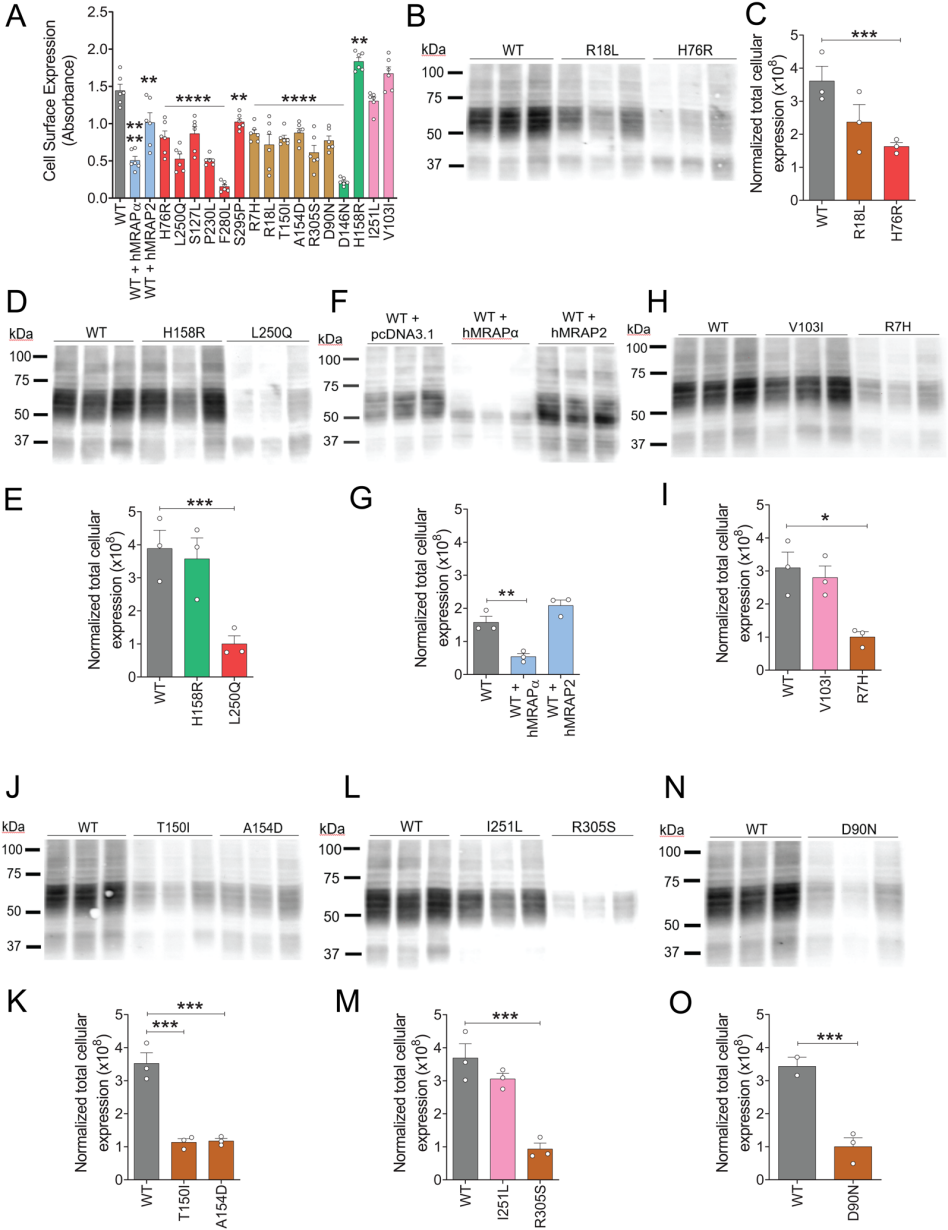
Cell-surface and total cellular HA-hMC4R expression for WT hMC4R compared with hMC4R variants and WT hMC4R co-expressed with either hMRAPα or hMRAP2 transiently transfected in HEK293 cells. We measured cell-surface hMC4R expression using live-cell ELISA on HEK293 cells following transient double transfections using DNAs for WT hMC4R + pcDNA3.1, hMRAPα or hMRAP2, or hMC4R variants + pcDNA3.1 (A). We performed three independent experiments with duplicates for each data point. We measured total hMC4R expression on cell lysates prepared from HEK293 cells following transient double transfections using DNAs for WT hMC4R + pcDNA3.1 (B–O), R18L + pcDNA3.1 (B, C), H76R + pcDNA3.1 (B, C), H158R + pcDNA3.1 (D, E), L250Q + pcDNA3.1 (D, E), WT hMC4R + hMRAPα (F, G), WT hMC4R + hMRAP2 (F, G), V103I + pcDNA3.1 (H, I), R7H + pcDNA3.1 (H, I), T150I + pcDNA3.1 (J, K), A154D + pcDNA3.1 (J, K), I251L + pcDNA3.1 (L, M), R305S + pcDNA3.1 (L, M) and D90N + pcDNA3.1 (N, O). We performed three independent experiments with one sample for each separate transfection per experiment. We electrophoretically separated total cellular proteins by SDS-PAGE, transferred them to membranes, and then detected HA-hMC4R protein using anti-HA antibody and western blotting. hMC4R protein separated into a spectrum of band sizes due to complex N-linked glycosylation (Kay *et al.* 2013*b*, Gillyard *et al.* 2019). The signals from all of these bands for each hMC4R variant on a western blot were combined and reported as total HA-hMC4R cellular protein normalized to total protein. We showed the data as mean ± s.e.m. We performed one-way ANOVA and Dunnett's posthoc analysis to determine significant differences compared to WT hMC4R. ^*^P < 0.05; ^**^P < 0.01; ^***^P < 0.001; ^****^P < 0.0001.

We showed significantly reduced cell-surface hMC4R protein for H76R (reduced by 39.6%; *P* < 0.0001), L250Q (reduced by 63.6%; *P* < 0.0001), and WT hMC4R co-expressed with hMRAPα (reduced by 64.9%; *P* < 0.0001) compared to WT hMC4R (Fig. 6A). In contrast, we showed significantly increased cell-surface hMC4R expression for H158R (increased by 27.04%, *P* < 0.01) compared to WT hMC4R (Fig. 6A). This increased H158R cell-surface expression likely accounts for H158R constitutive activity as well as H158R responsiveness to α-MSH-induced CRE-β-Gal reporter gene activity. However, the 40–65% reduced cell-surface hMC4R expression for obesogenic constitutively active hMC4R variants is unlikely to explain their impaired α-MSH-induced CRE-β-Gal reporter gene activity. All non-constitutively active LoF hMC4R variants studied similarly showed significantly reduced cell-surface expression (Fig. 6A and Supplementary Table 7), and yet, they remained responsive to α-MSH-induced CRE-β-Gal reporter gene activity (Fig. 2). Cell-surface expression for hMC4R variants that reduce the risk of developing obesity, V103I and I251L, was similar to that of WT hMC4R (Fig. 6A), confirming previously published data (see Supplementary Table 1 and (Brouwers *et al.* 2021)).

We also observed significantly reduced total hMC4R cellular expression for H76R (reduced by 54.9 %; *P* = 0.0235) (Fig. 6B and C), L250Q (reduced by 74.2 %; *P* = 0.0123) (Fig. 6D and E), and WT hMC4R co-expressed with hMRAPα (reduced by 65.8 %; *P* = 0.0052) (Fig. 6F and G), compared to WT hMC4R. Non-constitutively active LoF hMC4R variants also exhibited significantly reduced total hMC4R expression in comparison with WT hMC4R (Fig. 6H, I, J, K, L, M, N, and O and Supplementary Table 7). Similar to the cell-surface expression for hMC4R, total cellular hMC4R expression for V103I (Fig. 6H and I) and I251L did not differ from WT hMC4R (Fig. 6L and M), confirming previous reports (see Supplementary Table 1 and (Brouwers *et al.* 2021)).

### Evidence supporting intracellular constitutive AC and CRE-β-Gal reporter gene activity for obesogenic and non-obesogenic constitutively active hMC4R variants and WT hMC4R co-expressed with hMRAPα

**Figure 7:**
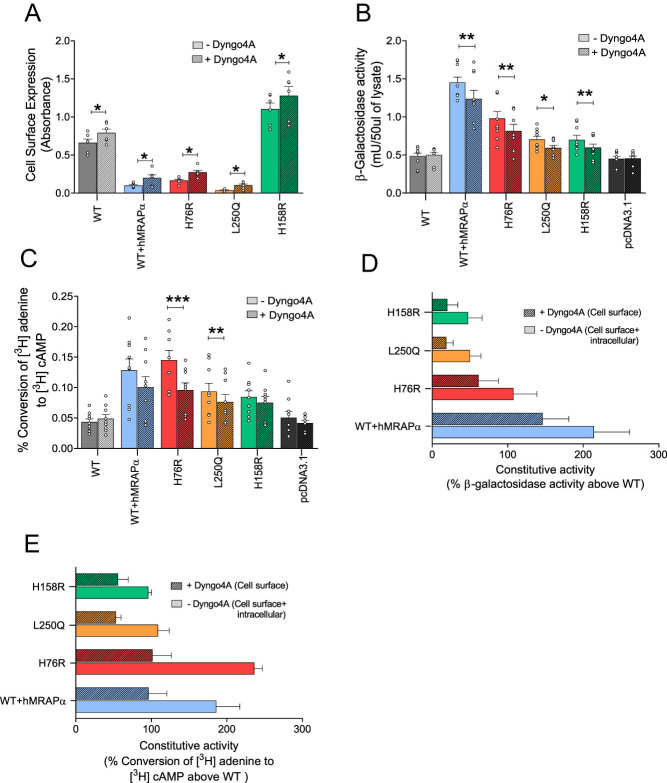
Effects of pre-treatment with Dyngo4a on cell-surface hMC4R expression, basal AC activity, and basal CRE-β-Gal reporter gene activity for WT hMC4R, hMC4R variants, and WT hMC4R co-expressed with hMRAPα. HEK293 cells were transiently double transfected with DNAs for WT hMC4R + pcDNA3.1, hMRAPα or hMRAP2, or hMC4R variants + pcDNA3.1 for cell-surface hMC4R expression and basal AC activity. HEK293 cells were transiently triple transfected with DNAs, including CRE-β-Gal reporter gene for WT hMC4R + pcDNA3.1, hMRAPα or hMRAP2, or hMC4R variants + pcDNA3.1 for basal CRE-β-Gal reporter gene activity. Following pre-treatment of cells with vehicle (control) or Dyngo4a, we measured cell-surface hMC4R expression by live-cell ELISA (A), basal CRE-β-Gal reporter gene activity (B), and basal AC activity (C). We pooled data from duplicate/triplicate wells of three independent experiments and showed them as mean ± s.e.m. We performed one-way ANOVA and determined significant differences as follows: ANOVA for panels A, B, and C, *P* < 0.0001. Tukey’s multiple comparison test in panel A for WT hMC4R vs WT hMC4R/hMRAPα, WT hMC4R vs H76R, WT hMC4R vs L250Q, and WT hMC4R vs H158R, with or without Dyngo4a, *P* < 0.0001. Tukey’s multiple comparison test in panel B for WT hMC4R vs WT hMC4R/hMRAPα with or without Dyngo4a, *P* < 0.0001 and WT hMC4R vs H76R without Dyngo4a, *P* < 0.0001, and with Dyngo4a, *P* < 0.05. Tukey’s multiple comparison test in panel C for WT hMC4R vs WT hMC4R/hMRAPα and WT hMC4R vs H76R with Dyngo4a, *P* < 0.0001. The statistical significance shown on the graphs was determined using the non-parametric pairwise Student’s *t*-test between vehicle and Dyngo4a-treated cells. ^*^P < 0.05; ^**^P < 0.01; ^***^P < 0.001. We calculated the total constitutive activity by expressing hMC4R functional activity as a percentage of WT hMC4R activity following pre-treatment with vehicle or Dyngo 4a (D, E).

We hypothesized that constitutively active hMC4R variants expressed intracellular constitutive activation of AC and CRE-driven gene transcription. To investigate, we pre-treated cells with vehicle (control) or Dyngo4a to block hMC4R constitutive internalization (McDaniel *et al.* 2012). Dyngo4a action was validated by showing that Dyngo4a pre-treatment significantly increased cell-surface hMC4R protein expression for WT hMC4R (increased by 19.9 %; *P* = 0.0381), H76R (increased by 66.6 %; *P* = 0.0171), L250Q (increased by 85.6 %; *P* = 0.012), H158R (increased by 15.9 %; *P* = 0.0251), and WT hMC4R co-expressed with hMRAPα (increased by 102.7 %; *P* = 0.0495) compared to vehicle pre-treatment (Fig. 7A).

Pre-treatment with Dyngo4a significantly reduced constitutive CRE-β-Gal reporter gene activity for H76R (reduced by 16.7 %; *P* = 0.0074), L250Q (reduced by 15.8 %; *P* = 0.0109), H158R (reduced by 14.7%; *P* = 0.0025), and WT hMC4R co-expressed with hMRAPα (reduced by 14.8 %; *P* = 0.0079) compared to vehicle pre-treatment (Fig. 7B). These data support intracellular constitutive CRE-β-Gal reporter gene activity for all these variants but not WT hMC4R. However, CRE-β-Gal reporter gene activity remained significantly increased for H76R (*P* = 0.0044) and WT hMC4R co-expressed with hMRAPα (*P* < 0.0001) compared to WT hMC4R with Dyngo4a pre-treatment. Pre-treatment with Dyngo4a also significantly reduced constitutive AC activity for H76R (reduced by 34.1 %; *P* = 0.0002) and L250Q (reduced by 18.2 %; *P* = 0.0063) and induced a trend toward reduced constitutive AC activity for H158R and WT hMC4R co-expressed with hMRAPα compared to vehicle (Fig. 7C). Overall, our data support cell-surface and intracellular constitutive AC and CRE-β-Gal reporter gene activity for H76R, L250Q, and H158R variants as well as WT hMC4R co-expressed with hMRAPα. Dyngo4a pre-treatment also revealed that intracellular hMC4R accounted for approximately 25–60% of total constitutive activity (Fig. 7D and E). Further studies are required to confirm this since Dyngo4a may have only partially blocked internalization in this study.

Based on these data, we hypothesized that (i) α-MSH binds cell-surface WT hMC4R and non-obesogenic constitutively active hMC4R variant (H158R) to induce AC activity near the cell surface and internalization of hMC4R results in α-MSH-induced intracellular hMC4R CRE-β-Gal transcription activity and (ii) α-MSH binds cell-surface obesogenic constitutively active hMC4R variants (H76R and L250Q) and hMRAPα-induced constitutively active WT hMC4R to induce AC activity near the cell surface. Then, either α-MSH does not internalize these hMC4R variants or it internalizes these variants to an intracellular location that does not support α-MSH-induced CRE-driven transcription.

## Discussion

We are the first to identify a unique signaling profile for H76R and L250Q variants that includes constitutive activity for AC, CRE-driven transcription, and Ca^2+^ mobilization, with WT-like basal pERK1/2 activity and markedly reduced total and cell-surface hMC4R protein expression compared to WT hMC4R. Importantly, we showed the obesogenic constitutively active signaling profile includes impaired α-MSH-induced CRE-driven transcription but not impaired α-MSH-induced AC, [Ca^2+^]_i_, or pERK1/2 signaling. This signaling profile was unique to obesogenic constitutively active hMC4R variants since neither impaired α-MSH-induced CRE-driven transcription nor reduced cell-surface and total hMC4R expression was observed for H158R, a constitutively active hMC4R variant associated with overweight but not obesity.

Based on this discovery, we hypothesize that α-MSH-induced hMC4R-CRE-driven transcription is required for body weight regulation. Further, we hypothesize that constitutive AC activity, CRE-driven transcription, and [Ca^2+^]i mobilization are not able to prevent human obesity, at least in the presence of impaired α-MSH-induced CRE-driven transcription. Therefore, we propose that *in vitro* α-MSH-induced CRE-driven reporter gene activity could be a ‘gold standard’ assay in future studies to determine hMC4R variant functionality. First, however, we suggest that the CRE-luciferase reporter plasmid be directly compared with CRE-β-Gal reporter plasmid, as these reporter activity measurements do not appear equivalent. The CRE-β-Gal reporter appears more sensitive than CRE-luciferase reporter for detecting hMC4R LoF *in vitro* (see Supplementary discussion). We predict that α-MSH-induced hMC4R coupling to an optimized CRE-driven reporter gene will be a reliable method to determine whether a variant exhibits hMC4R LoF associated with obesity. Further, we propose α-MSH-induced CRE-driven reporter gene activity be the preferred high-throughput screen to discover potential safe and effective anti-MC4R signaling therapeutics targeting obesity.

We showed that the unique obesogenic constitutively active hMC4R signaling profile was shared with hMRAPα-induced hMC4R constitutive activity. We previously reported that hMRAPα and hMC4R heterologously expressed in HEK293 cells predominantly colocalized intracellularly in highly dynamic vesicles located in the endoplasmic reticulum and Golgi apparatus (Kay *et al.* 2015). These data support our new finding that hMC4R constitutive activity arises in part from intracellular receptors. If hMRAPα induced hMC4R constitutive activity *in vivo*, we hypothesize this would cause obesity. However, for this to happen, MRAPα and MC4R would need to colocalize in cells where MC4R functions to regulate body weight. Currently, there is no evidence of their colocalization in any cell type in any tissue in which they are co-expressed (Gardiner *et al.* 2002, Kay *et al.* 2013*a*).

Finally, our study raises many questions about how the intracellular MC4R network impacts energy homeostasis. Intracellular GPCR cellular location has recently emerged as critical to receptor function and disease pathology (Eichel & von Zastrow 2018, Jong *et al.* 2018, Weinberg & Puthenveedu 2019). Future studies are needed to examine MC4R signaling where ‘location bias’ of cell-surface and intracellular receptors is only beginning to be explored (Brouwers *et al.* 2021). Detailed functional studies of naturally occurring constitutively active hMC4R variants compared to WT hMC4R have the potential to significantly advance our understanding of the signaling mechanism hMC4R uses to regulate energy homeostasis.

## Supplementary Materials

Supplementary Material

Supplementary Figure 1

Supplementary Figure 2

Supplementary Figure 3

Supplementary Figure 4

Supplementary Table 1: Naturally occurring hMC4R variants selected for this study showing previously identified characteristics for each variant. BMI, body mass index; AC, adenylyl cyclase; RIA, radioimmunoassay.

Supplementary Table 2: List of primers generated for SOE cloning of HA-hMC4R variants.

Supplementary Table 3: Biochemical Reagents

Supplementary Table 4

Supplementary Table 5

Supplementary Table 6

Supplementary Table 7

## Declaration of interest

The authors declare no competing interests.

## Funding

Funding for this study was provided by the Maurice and Phyllis Paykel Trusthttp://dx.doi.org/10.13039/501100001518 and The University of Aucklandhttp://dx.doi.org/10.13039/501100001537 postgraduate student fund. The University of Aucklandhttp://dx.doi.org/10.13039/501100001537 awarded Rikus Botha and Shree Kumar PhD scholarships.

## Author contribution statement

KGM and RB were responsible for the overall experimental design. RB developed the constructs, performed the experiments, analyzed data, and wrote the manuscript. SSK performed experiments, analyzed data, prepared figures, and contributed to the writing of the manuscript. NLG provided expertise with live-cell ELISA methodology and contributed to writing the manuscript. KGM and SSK finalized data analysis and writing of the manuscript, which all authors then reviewed.
