# Supplementary material for: A unique melanocortin-4-receptor signaling profile for obesity-associated constitutively active variants
Source: J Mol Endocrinol. 2023 Jun 12;71(1):e230008. doi: 10.1530/JME-23-0008 (PMC10304906; doi:10.1530/JME-23-0008)
Supplement: Supplementary Material [file supplementary_material.pdf]

Supplementary Materials and Methods

Construction of HA-hMC4R variants

Each construct was cloned using three independent PCR reactions and six different primer pairs, two of which were unique to each point mutation **(Supplementary Table 2)**. PCR#1 used a generic 5' primer (HA-hMC4RForR1) targeting the pcDNA3.1 plasmid multiple cloning site upstream of the HA-epitope and a variant-specific 3' primer incorporating the target point mutation. PCR#2 used a generic 3' primer (HA-hMC4RRevR2) targeting the multiple cloning site downstream of the hMC4R stop codon and a variant-specific 5' primer incorporating the target point mutation. PCR#1 and #2 products were combined, DpnI treated to lyse the original HA-hMC4R template, and then used as template DNA in PCR#3. PCR#3 amplified full-length variant HA-hMC4R with 5'KpnI and 3' XhoI restriction sites encoded in HA-hMC4RForR3 and HA-hMC4RRevR3 primers. SOE PCR#1 and #2 were performed in a 30 µl reaction mix containing 1 ng of HA-hMC4R-WT pcDNA3.1 template DNA and iProof High Fidelity DNA Polymerase under the following conditions: denature (98°C, 1 min; 98°C, 5 s), anneal (62°C, 30 s), elongate (72°C, 10 s) for 35 cycles and then one final 7 min elongation at 72°C. PCR product from PCR#1 and #2 (8.2 µl of 30 µl PCR final volume) was DpnI digested in a final volume of 10 µl with 0.8 units in supplier recommended buffer at 37°C for 1 h. DpnI digested PCR#1 and #2 products were then pooled (1 µl of each) and used as template DNA for PCR#3 using the PCR conditions described above, except for annealing temperate being 63°C. Each HA-hMC4R variant was then subcloned into pcDNA3.1 and verified by sequencing and subsequent alignment to NM_005912.2.

Cell culture

The HEK293 cells originally sourced from ATCC, were authenticated for this study. The SNPs tested were 100% identical to ATCC clone CRL-12013. HEK293 were grown in

DMEM supplemented with 10% (v/v) NCS and 1% (v/v) P/S at 37°C under 5% CO_2. Mouse hypothalamic neuronal cells (GT1-7) were grown in DMEM supplemented with 10% (v/v) FCS and 1% (v/v) P/S at 37°C under 5% CO_2.

CRE-β-Gal reporter gene assay

3,500 HEK293 or 1,900 GT1-7 cells were seeded per well in 96-well plates and transfected 24 h later when the cells were ~50% confluent with a total of 85 ng plasmid DNA and 0.25 µl of Fugene 6. After an additional 72 h, cells were stimulated with increasing concentrations of α-MSH (0 M for basal activity) prepared in 200 µL DMEM + 0.1% Bovine Serum Albumin (BSA) for 6 h at 37°C under 5% CO_2. Following stimulation, cells were washed with 100 µL 1x PBS and lysed in 50 µl of 1x RLB for 20 min and then stored at -80 °C until assay. After thawing, 25 µl of lysate from each α-MSH stimulated well was transferred to a 96-well assay plate containing 25 µl of 1x RLB per well (1:2 final dilution of lysate) and β-Gal measured following the Promega β-Gal enzyme assay protocol. For basal activity wells, 50 µl of thawed lysate was transferred to a 96-well assay plate. Freshly prepared β-Gal standards (0-5 milliunits) prepared in 50 µL 1x RLB as per manufacturer instructions were also loaded onto the 96 well plates. All wells with either standards or lysates then received 50 µl of 2 x assay buffer (200 mM Sodium phosphate buffer- pH 7.3, 2 mM MgCl2, 100 mM β-ME, 1.33 mg/ml ONPG), and the plate was incubated at 37°C for 30 min for color development. The reaction was stopped with 150 µl of 1 M sodium carbonate, and the absorbance was read at 420 nm. Sample absorbance readings at 420 nm were converted to β-Gal activity per well using the β-Gal standards present on each plate to obtain β-Gal milliunits per 25 µl. This was multiplied by two to account for lysate dilution and yielded β-Gal activity per well of a 96-well plate. For basal activity, the β-Gal milliunits per 50 µl represent β-Gal activity per well.

AC assay

80,000 HEK293 or 75,000 GT1-7 cells were seeded per well in 24-well plates. After 48 h, each well at ~50% confluency was transfected with a total of 0.5 μg of plasmid DNA and 1.5 μl of Fugene 6. Following an additional 48 h, AC activity was measured as previously described (Kay et al. 2013a; Mountjoy, et al. 1999).

Pre-treatment of cells with PTX

4,500 HEK293 cells were seeded per well in 96-well plates and transfected 24 h later as described above**.** After ~54 h post-transfection, each well was pre-incubated for 18 h at 37°C with or without 50 ng/ml PTX. Following 72 h, cells were stimulated with increasing concentrations of α-MSH (0 M for basal activity) prepared in 200 μL DMEM + 0.1% BSA + 50 ng/ml PTX, or control buffer for 6 h at 37°C under 5% CO_2. We performed the CRE-β-Gal reporter gene assay described above.

Calcium assay

To study hMRAPα-induced hMC4R constitutive activity, either pcDNA3.1 (control) or hMRAPα was transiently expressed in HEK293 cells stably expressing WT hMC4R. For these experiments, 2.4 x 10^6 WT hMC4R stably transfected cells were seeded in 10 cm tissue culture plates and 24 h later they were transfected with 7.5 μg pcDNA3.1 or hMRAPα mixed with 45 μl of Fugene 6. 24 h later the cells were seeded in a T75 tissue culture flask. After a further 48 h, the calcium assay was performed.

Pre-treatment of cells with ESI-09, an EPAC1/2 **inhibitor.**

To determine whether hMC4R variant mediated CRE-β-Gal reporter gene activity was mediated by EPAC1/2, cells were pre-treated for 30 min with 190 μL DMEM + 0.1% BSA+ 20 μM ESI-09 or DMSO (control), before spiking with α-MSH concentrations, followed by a 6 h incubation. To determine whether hMC4R mobilization of intracellular calcium was EPAC1/2 mediated, cells were pre-treated with 210 μL calcium loading buffer + 10 μM ESI-09 or DMSO (control) for 30 min before α-MSH stimulation.

Lysate generation for western blots

80,000 HEK293 cells were seeded per well in 24-well plates. The cells were ~50% confluent after 48 h and then cells in each well were transfected with a total of 0.5 μg plasmid DNA and 1.5 μl of Fugene 6. After an additional 48 h, cells were incubated in 500 mL DMEM + 0.1% BSA for 1 h at 37°C under 5% CO_2 followed by lysing the cells in each well with 55 μl of PBS + 1% n-Dodecyl-β-D-maltoside + Complete mini Protease inhibitors and PhosStop, for 15 min with the plate kept on ice. Cells were then scraped using mini-cell scrapers and the lysates were transferred into eppendorf tubes, briefly vortexed and incubated on ice for 15 min to complete lysis. The lysates were then centrifuged at 4000 x g at 4°C for 10 min before the supernatants were transferred into new eppendorf tubes and the lysate samples stored at -20°C. The protein concentration for each lysate was quantitated in duplicate using a Bio-Rad DC protein assay kit.

Western blots

Lysates were thawed, vortexed and briefly centrifuged before being sonicated 3x for 10 s (with 10 s intervals on ice between sonication). Lysates were again vortexed and briefly centrifuged before removing 20 μg of lysate which was combined with 2x Laemmli buffer + 5% freshly added β-ME and made up to a 12 μl final volume with MilliQ water. Lysates were

heated to 65°C for 10 min and then loaded into 10% precast polyacrylamide TGX strain-free gels containing the trihalo-compound, trichloroethano, which covalently binds tryptophan residues of proteins under UV exposure (Gilda and Gomes 2013; Ladner, et al. 2004; Moritz 2017). At least one lane on each gel was loaded with 2.5 μl Precision Unstained Standard to estimate the molecular mass of specific bands. Gels were electrophoresed at 100 V for 3 min and then 150 V for 48 min using a Mini-PROTEAN Tetra Cell system (BioRad Laboratories, PA, USA). Gels were removed from cassettes and the stain-free system activated through a 1 min UV light exposure and an image captured of total protein loaded on the gel using ImageLab 5.1 software and a ChemiDoc (BioRad Laboratories). The proteins on the gel were then electrophoretically transferred to a membrane using a Trans-Blot Turbo system and an in-house optimised protocol (25 V, 2.5 A and 4 min running time). Briefly, 0.2 μM PVDF membrane was cut to match the gel size, activated in 100% methanol for 3 s and then equilibrated in Towbin transfer buffer (25 mM Tris, 192 mM glycine, 20% methanol) for 30 min. Simultaneously, Trans-Blot Turbo Mini transfer pads were removed from their package/buffer and incubated for 30 min in Towbin transfer buffer. The transfer stack was then assembled as per the Bio-Rad recommended protocol before transferring protein lysates using the Transblot Turbo transfer.

Post transfer, PVDF blots were washed two times for 5-minutes in TBS-T post-transfer and non-specific sites then blocked through incubation with Tris buffered Saline (TBS; 200mM Tris, 1.37M NaCl, pH7.5) + 0.1% (v/v) Tween 20 (TBS-T) + 5% BSA for 1.5-hours. PVDF blots were then incubated overnight at 4°C with 1:16k rabbit anti-pERK1/2 monoclonal antibody prepared in TBS-T + 5% BSA. PVDF blots followed by four washes for 5-minutes in TBS-T and incubation with 1:40k goat anti-rabbit HRP conjugated secondary antibody prepared in TBS-T + 5% BSA for 2-hours at RT. PVDF blots were then washed three times

for 10-minutes in TBS-T before development with Clarity ECL. PVDF blot chemiluminescent signals were visualized on a ChemiDoc and then the blots were stored at 4°C in TBS-T until required for stripping. Blots were subsequently washed two times for 5-minutes in TBS-T before stripping using Abcam's harsh stripping protocol (Abcam, 2017). Blots were then washed two times for 5-minutes in TBS-T before blocking for 1.5-hours in TBS-T + 5% BSA at RT. PVDF blots were then incubated overnight at 4°C with 1:10k rabbit anti-ERK1/2 monoclonal antibody prepared in TBS-T + 5% BSA. PVDF blots were then washed four times for 5-minutes in TBS-T before incubation with 1:40k goat anti-rabbit HRP conjugated secondary antibody prepared in TBS-T + 5% BSA for 2-hours at RT. PVDF blots were then washed three times for 10-minutes in TBS-T before chemiluminescent signal development with Clarity ECL. Chemiluminescent signals were visualized on a ChemiDoc (Bio-Rad Laboratories, 139 Philadelphia, PA, USA) and analyzed using ImageLab version 5.2.1 as per the Bio-Rad recommended protocol. Intensities of specific bands obtained by chemiluminescence were normalized to the entire UV intensity of the corresponding sample, representing the total protein content for the sample loaded on the membrane. The lanes were detected automatically for both the phosphorylated and total protein chemiluminescence signal on the membrane and lanes were manually adjusted on the software when required to include relevant regions of signal data only. Custom band detection was used to separately detect pERK1/2 / ERK1/2 isoforms according to Precision Plus protein standards. Background subtraction was enabled and the disk size was reduced to 3-7mm for all lanes as required to ensure consistent background subtraction for both the phosphoprotein and total protein chemiluminescent blots. The sums of pERK1 + pERK2 and ERK1 + ERK2 were used for analysis.

ERK1/2 and pERK1/2 protein expression: Western blots

Cell lysates and western blots were prepared. Post transfer, PVDF blots were washed 2x for 5 min in Tris-buffered Saline (TBS; 200mM Tris, 1.37M NaCl, pH7.5) + 0.1% (v/v) Tween 20 (TBS-T) and non-specific sites were then blocked through incubation with TBS-T + 5% BSA for 1.5 h. PVDF blots were incubated overnight at 4°C with a 1:16k rabbit anti-pERK1/2 monoclonal antibody prepared in TBS-T + 5% BSA to detect signal. The blots were then washed 4x for 5 min in TBS-T and incubation with 1:40k goat anti-rabbit HRP conjugated secondary antibody prepared in TBS-T + 5% BSA for 2 h at RT. PVDF blots were then washed 3x for 10mins in TBS-T before development with Clarity ECL. We visualized the PVDF blot chemiluminescent signals on a ChemiDoc, and the blots were then stored at 4°C in TBS-T until required for stripping. Next, we washed the blots 2x for 5 min in TBS-T before stripping with Abcam's harsh stripping protocol (Abcam.com/protocols). We then washed the blots 2x for 5min in TBS-T before blocking for 1.5 h in TBS-T + 5% BSA at RT. PVDF blots were incubated overnight at 4°C with a 1:10k rabbit anti-ERK1/2 monoclonal antibody prepared in TBS-T + 5% BSA. We then washed the PVDF blots 4x for 5mins in TBS-T before incubation with 1:40k goat anti-rabbit HRP conjugated secondary antibody prepared in TBS-T + 5% BSA for 2 h at RT. Following this, we washed the PVDF blots 3x for 10min in TBS-T before chemiluminescent signal development with Clarity ECL. Chemiluminescent signals were visualized on ChemiDoc (Bio-Rad Laboratories, 139 Philadelphia, PA, USA) and analyzed using ImageLab version 5.2.1 as per Bio-Rad protocol. Intensities of specific bands obtained by chemiluminescence were normalized to the entire UV intensity of the corresponding sample, thus representing the total protein content for the sample loaded on the membrane (Vigelso, et al. 2015). The lanes were detected automatically for both the phosphorylated and total protein signal on the membrane and then manually adjusted on the software when required to include relevant regions of signal data only. Precision Plus protein standards were used as custom band detection to separately detect

pERK1/2 / ERK1/2 isoforms. Background subtraction was enabled, and disk size was reduced to 3-7 mm for all lanes as required to ensure consistent background subtraction for both the phosphoprotein and total protein chemiluminescent blots. We summed pERK1 + pERK2 and ERK1 + ERK2 for analysis.

Total cellular HA-hMC4R protein expression: Western blot

Post protein transfer, PVDF membranes were washed 2x for 5 min in 30 mL TBS-T, and non-specific antibody binding sites were blocked through incubation of the membranes with 50 mL 5% low-fat milk powder + TBS-T for 1.5 h. The PVDF membranes were incubated overnight at 4°C with 2.5 mL 1:5k mouse anti-HA.11 monoclonal antibody prepared in TBS-T + 1% low-fat milk powder, washed 4x for 5 min in 30 mL TBS-T before incubation with 1:10k sheep anti-mouse horseradish peroxidase-conjugated secondary antibody prepared in TBS-T + 1% low-fat milk powder, for 2 h at RT. The PVDF membranes were then washed 3x for 10 min in 30 mL TBS-T before developing a signal with Clarity ECL. PVDF blot chemiluminescent signals were visualized on a ChemiDoc and analyzed using ImageLab, as described in 2.10 with the following exceptions. The lanes were detected automatically for both chemiluminescence signal and stain-free protein (image captured post-transfer), and lanes were manually adjusted on the software when required to include relevant regions of signal data. One single band was added to each lane and the band resized to span the full spectrum of observed total expression in that lane (band spanning ~35 kDa to ~250 kDa). This spectrum of band sizes is due to complex N-linked glycosylation (Kay et al. 2013b). Background subtraction was enabled, and the disk size was reduced to 5-8mm for all lanes as required to ensure consistent background subtraction for both the chemiluminescent and stain-free blots. Total protein normalization data was then transferred into GraphPad Prism 7.0 for analysis.

Cell surface HA-hMC4R protein expression: ELISA

15,000 HEK293 cells were seeded per well in 24-well plates pre-coated with 0.2 mg/ml poly-L-lysine. The cells were ~50% confluent after 48 h when they were transfected with a total of 0.5 μg plasmid DNA and 1.5 μl of Fugene 6. After an additional 48 h, we placed the plate on ice, aspirated the media, and incubated the cells with 300 μl per well of 1:1000 mouse anti-HA.11 monoclonal antibody (prepared in DMEM + 0.25 mM HEPES) for 1 h at 4°C with gentle agitation. With the plate kept on ice, cells were washed twice with 500 μL ice-cold DMEM before they were fixed in 500 μL per well of 2% PFA (pH 7.4) for 10 min at 4°C with gentle agitation. The use of 2% PFA and not 4% PFA for non-permeabilized cells was adapted from a previous method used to immuno-stain cell surface melanocortin receptors for confocal microscopy (Kay et al. 2013a). Previously, we observed some internalization of cell surface receptors with 4% PFA but not with 2% PFA. Cells were then washed twice with 500 μL 1x PBS for 5 min at 4°C with gentle agitation before incubation with 0.3 mL per well of 1:20k sheep anti-mouse horseradish peroxidase (HRP) conjugated secondary antibody (prepared in 1x PBS + 1% low-fat milk powder) for 2 h at 4°C with gentle agitation. Cells were then washed 3x with 500 μL 1x PBS for 10 min before incubation with 200 μl of BM Blue POD Substrate per well for 10 min at room temperature for color development. The reaction was stopped by adding 200 μl per well of 10% sulfuric acid. Then 300 μl of reaction mix was transferred to a 96-well plate, and the absorbance was read at 450 nm and 690 nm using a PHERAstar FS plate reader (BMG Labtech, Germany). Cell surface expression was calculated for each well by subtracting 690 nm background absorbance from the 450 nm absorbance, then subtracting averaged absorbance from HRP binding to HEK293 cells transfected with empty pcDNA3.1 alone.

Pre-treatment of cells with Dyngo4a

To determine whether Dyngo4a could inhibit constitutive hMC4R internalization, we measured hMC4R protein expression at the cell surface following treatment of cells with vehicle or Dyngo4a. 15,000 HEK293 cells were seeded per well in 24-well plates pre-coated with 0.2 mg/ml Poly-L-Lysine and transfected 48 h later as described in 2.11. Then 48 h post-transfection, each well was pre-incubated for 2 h at 37°C under 5% CO2 in fresh DMEM + NCS + P/S with or without 30 μM Dyngo4a or DMSO control (DMSO was diluted 1:5000 to match the final DMSO concentration in Dyngo4a treated wells). Following this, cell surface HA-hMC4R protein expression was determined by ELISA.

To determine whether AC constitutive activity arises from either cell surface or intracellular receptors for hMC4R variants and hMRAPα-induced constitutively active hMC4R, we seeded 120,000 HEK293 cells per well in 24-well plates and transfected the cells 48 h later as described in 2.6. Approximately 48 h post-transfection, cells were equilibrated for 2 h at 37ºC with [^{3}H] adenine (2.5μCi/ml) in the presence of either 30μM Dyngo4a or DMSO control. The media was then removed, cells washed with 1x PBS, and increasing concentrations of α-MSH (0 M for basal activity) prepared in 495 μL of fresh DMEM + 0.1% BSA + 0.5 mM IBMX. Immediately following incubation medium application, wells were spiked with 5 μL of Dyngo4a or DMSO control to reach 30 μM final concentration. The cells were incubated for a further 1 h at 37°C under 5% CO_2 before we performed the AC assay.

To determine whether CRE-β-Gal reporter gene constitutive activity arises from either cell surface or intracellular receptors for hMC4R variants and hMRAPα-induced constitutively active hMC4R, we seeded 4,500 HEK293 cells per well in 96-well plates and transfected 24 h later. Approximately 48 h post-transfection, the media was removed and cells were

incubated with DMEM + 0.1% BSA + either 30mM Dyngo4a or DMSO control for 6 h at 37°C under 5% CO_2. The media was then removed, cells washed with 1x PBS, lysed, and β-Gal reporter gene activity measured.

Statistical analysis

GraphPad Prism 7.0 software was used to generate graphs and to perform statistical analyses. For basal AC, CRE-β-Gal reporter gene and calcium assays, raw AC, CRE-β-Gal reporter gene, or $[Ca^{2+}]_i$ data were pooled from three independent experiments. We performed One-Way ANOVA and Dunnett's post-hoc test to determine significance. For α-MSH induced responses, raw AC, CRE-β-Gal reporter gene, or $[Ca^{2+}]_i$ data were pooled from three independent experiments and normalized to the minimum and maximum response best-fit values for WT hMC4R. Sigmoidal concentration-response curves were then fitted to compare maximum, minimum, or EC_{50} values between WT hMC4R and hMC4R variants. Statistical significance was determined using the non-parametric sum of squares f-test. The span from baseline to maximum response was determined in Prism for each independent experiment when the AC, CRE-β-Gal reporter gene, or $[Ca^{2+}]_i$ baseline was significantly increased or decreased compared to WT hMC4R or untreated cells. To determine if the α-MSH-induced maximum response differed from WT hMC4R or untreated cells, the mean ± SEM span for three independent experiments were tested for significance using a paired student 't' test.

For basal and ligand stimulated pERK, phosphoprotein normalized to respective total protein data from three independent assays were compared and significance was determined using both two-way ANOVA and a non-parametric t-test.

Cell-surface HA-hMC4R protein expression data from three independent ELISA experiments plotted as absorbance at 450 nm minus absorbance at 690 nm, minus background absorbance (obtained from empty pcDNA3.1 vector-transfected wells) were compared. For total cellular HA-hMC4R protein expression, normalized data from three independently generated lysates analyzed on the same western blot were compared. Significance was determined using One-Way ANOVA and Dunnett's Post-Hoc test.

For Dyngo4a inhibition experiments, data from three independent experiments were compared and significance determined using both two-way ANOVA and a non-parametric student t-test.

Supplementary Discussion

Using the CRE-β-Gal reporter, we showed complete impairment of α-MSH concentration-induced sigmoidal response for H76R. Gillyard *et al*. using the CRE-Luciferase reporter showed partial impairment that retained an agonist-induced sigmoidal response for H76R (Gillyard, et al. 2019). Also, using the CRE-Luciferase reporter assay, Gillyard *et al.* (Gillyard et al. 2019) failed to detect an impaired agonist-induced response for the R305S variant. We showed here an impaired α-MSH-induced response for R305S using the CRE-β-Gal reporter. Furthermore, we or others (**Supplementary Table 1**) have shown impaired α-MSH induced CRE-driven reporter gene activity for all of the obesogenic hMC4R variants studied here, except R7H and R18L. No study has identified any impaired intracellular signaling for R7H and R18L (**Supplementary Table 1**). Therefore, we question whether individuals expressing these variants are truly obese? They presented with a BMI of 30 and no further phenotyping to support obesity.

Surprisingly, the R7H and R18L variants exhibited significantly increased α-MSH induced CRE-b-Gal reporter gene activity (**Figure 2I, 2L**) and significantly increased α-MSH induced AC activity (**Figure 3I, 3L**) respectively. This was despite both variants exhibiting significantly decreased cell surface and total hMC4R protein expression compared with WT hMC4R. We predict α-MSH regulation of R7H and R18L receptor internalization and trafficking to intracellular locations may differ from WT hMC4R, and receptor location bias may account for the signaling differences. Therefore, enhanced R7H CRE-driven reporter activity and enhanced R18L AC activity may reflect increased and decreased respectively, trafficking to endosomes.

References

Chen W, Shields TS, Stork PJS & Cone RD 1995 A colorimetric assay for measuring activation of Gs- and Gq- coupled signaling pathways. *Analytical Biochemistry* **226** 349-354.

Dzhura I, Chepurny OG, Kelley GG, Leech CA, Roe MW, Dzhura E, Afshari P, Malik S, Rindler MJ, Xu X, et al. 2010 Epac2-dependent mobilization of intracellular Ca(2)+ by glucagon-like peptide-1 receptor agonist exendin-4 is disrupted in beta-cells of phospholipase C-epsilon knockout mice. *Journal of Physiology* **588** 4871-4889.

Fitzgerald LR, Mannan IJ, Dytko GM, Wu HL & Nambi P 1999 Measurement of responses from Gi-, Gs-, or Gq-coupled receptors by a multiple response element/cAMP response element-directed reporter assay. *Analytical Biochemistry* **275** 54-61.

Gilda JE & Gomes AV 2013 Stain-Free total protein staining is a superior loading control to beta-actin for Western blots. *Analytical Biochemistry* **440** 186-188.

Gillyard T, Fowler K, Williams SY & Cone RD 2019 Obesity-associated mutant melanocortin-4 receptors with normal Galphas coupling frequently exhibit other discoverable pharmacological and biochemical defects. *Journal of Neuroendocrinology* **31** e12795.

Glas E, Muckter H, Gudermann T & Breit A 2016 Exchange factors directly activated by cAMP mediate melanocortin 4 receptor-induced gene expression. *Sci Rep* **6** 32776.

Kay EI, Botha R, Montgomery JM & Mountjoy KG 2013a hMRAPalpha increases alphaMSH induced hMC1R and hMC3R functional coupling and hMC4R constitutive activity. *Journal of Molecular Endocrinology* **50** 203-215.

Kay EI, Botha R, Montgomery JM & Mountjoy KG 2013b hMRAPalpha specifically alters hMC4R molecular mass and N-linked complex glycosylation in HEK293 cells. *Journal of Molecular Endocrinology* **50** 217-227.

Kumar SS, Ward ML & Mountjoy KG 2021 Quantitative high-throughput assay to measure MC4R-induced intracellular calcium. *Journal of Molecular Endocrinology* **66** 285-297.

Ladner CL, Yang J, Turner RJ & Edwards RA 2004 Visible fluorescent detection of proteins in polyacrylamide gels without staining. *Analytical Biochemistry* **326** 13-20.

Manson ME, Corey DA, Rymut SM & Kelley TJ 2011 beta-arrestin-2 regulation of the cAMP response element binding protein. *Biochemistry* **50** 6022-6029.

Moritz CP 2017 Tubulin or Not Tubulin: Heading Toward Total Protein Staining as Loading Control in Western Blots. *Proteomics* **17** 1600189.

Mountjoy KG, Willard DH & Wilkison WO 1999 Agouti antagonism of melanocortin-4 receptor: greater effect with desacetyl-alpha-melanocyte-stimulating hormone (MSH) than with alpha-MSH. *Endocrinology* **140** 2167-2172.

Vallejo AN, Pogulis RJ & Pease LR 2008 PCR Mutagenesis by Overlap Extension and Gene SOE. *CSH Protoc* **2008** pdb prot4861.

Vigelso A, Dybboe R, Hansen CN, Dela F, Helge JW & Guadalupe Grau A 2015 GAPDH and beta-actin protein decreases with aging, making Stain-Free technology a superior loading control in Western blotting of human skeletal muscle. *J Appl Physiol (1985)* **118** 386-394.

Villasenor R, Kalaidzidis Y & Zerial M 2016 Signal processing by the endosomal system. *Current Opinion in Cell Biology* **39** 53-60.
