# Supplementary material for: A unique melanocortin-4-receptor signaling profile for obesity-associated constitutively active variants
Source: J Mol Endocrinol. 2023 Jun 12;71(1):e230008. doi: 10.1530/JME-23-0008 (PMC10304906; doi:10.1530/JME-23-0008)
Supplement: Supplementary Figure 1 [file supplementary_figure_1.pdf]

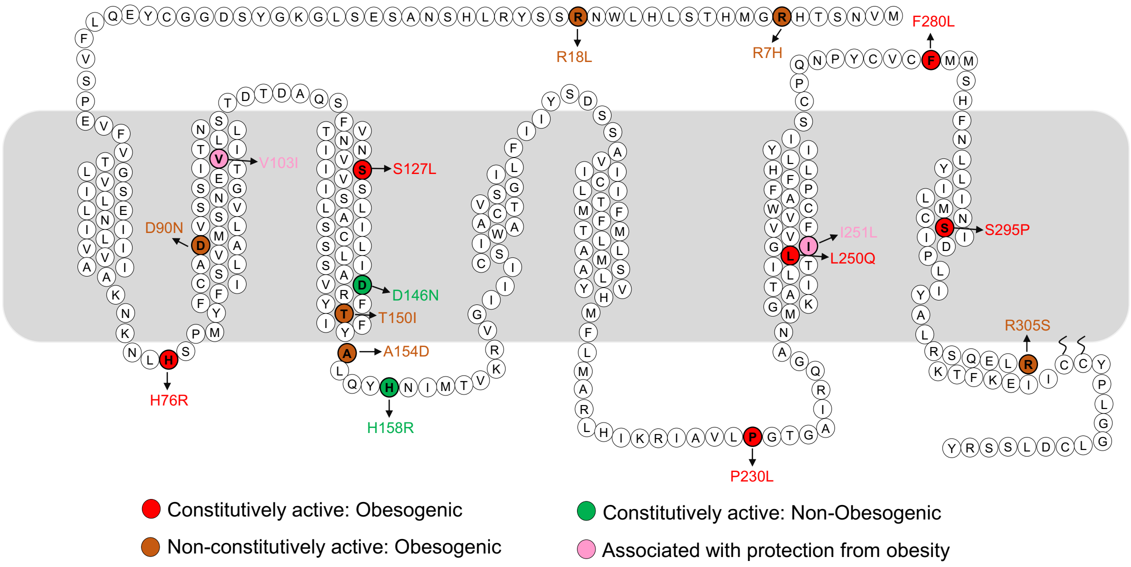

Supplementary Figure 1. Schematic of hMC4R showing locations of variants studied. The shaded area represents the plasma membrane. The transmembrane boundaries were set according to Kleinau *et al*. (Kleinau, et al. 2020).

Kleinau G, Heyder NA, Tao YX & Scheerer P 2020 Structural Complexity and Plasticity of Signaling Regulation at the Melanocortin-4 Receptor. *International Journal of Molecular Sciences* **21** 5728.
