# Supplementary material for: A unique melanocortin-4-receptor signaling profile for obesity-associated constitutively active variants
Source: J Mol Endocrinol. 2023 Jun 12;71(1):e230008. doi: 10.1530/JME-23-0008 (PMC10304906; doi:10.1530/JME-23-0008)
Supplement: Supplementary Figure 2 [file supplementary_figure_2.pdf]

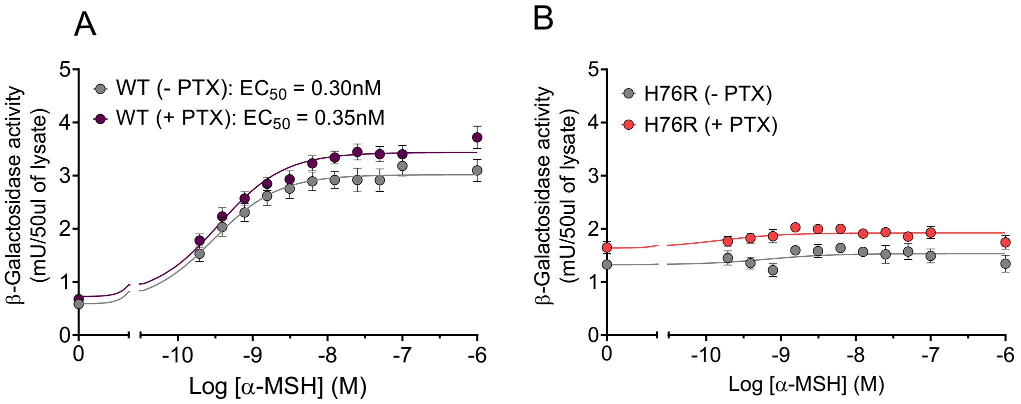

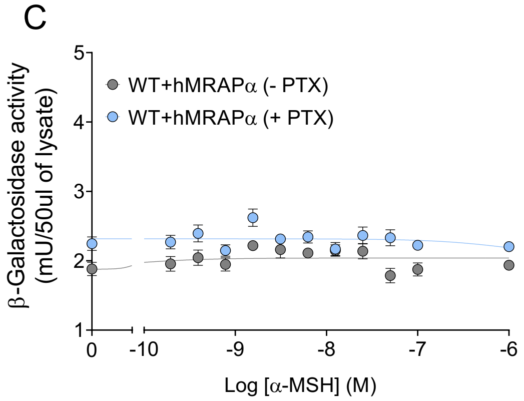

D

Variant -/+ PTX	Baseline -/+ PTX P value	Maximum -/+ PTX P value	Log EC_{50} -/+ PTX P value
WT	0.4637	0.0182	0.9874
H76R	0.0176		
WT+hMRAP α	< 0.0001		

Supplementary Figure 2. PTX pre-treatment significantly enhanced maximum α-MSH induced CRE-β-Gal reporter gene activity for WT hMC4R and constitutive CRE-β-Gal reporter activity for H76R and WT hMC4R co-expressed with hMRAPα. The CRE-β-Gal reporter gene assays were performed with transient triple transfections in HEK293 cells using DNAs for CRE-β-Gal reporter gene, WT hMC4R + pcDNA3.1 (**A**), H76R + pcDNA3.1 (**B**), and WT hMC4R + hMRAPα (**C**). α-MSH concentration-response curves were fitted to raw data for CRE-β-Gal reporter gene activity per well using GraphPad Prism. Summary of significance for comparative CRE-β-Gal reporter gene activity best-fit curve fitting for vehicle versus PTX (**D**). We pooled data from three independent experiments and data are shown as mean ± SEM. The non-parametric sum of squares f-test was used to determine significance for parameters derived from curve fitting. The statistical significance for the maximum constitutive response for H76R (**B**) and WT hMC4R co-expressed with hMRAPα (**C**) was determined by averaging CRE-β-Gal reporter gene activity across all α-MSH concentrations for pre-treatment with vehicle or PTX and tested the difference between vehicle and PTX responses for significance using pairwise Student t test (**D**).
