# Supplementary material for: A unique melanocortin-4-receptor signaling profile for obesity-associated constitutively active variants
Source: J Mol Endocrinol. 2023 Jun 12;71(1):e230008. doi: 10.1530/JME-23-0008 (PMC10304906; doi:10.1530/JME-23-0008)
Supplement: Supplementary Figure 3 [file supplementary_figure_3.pdf]

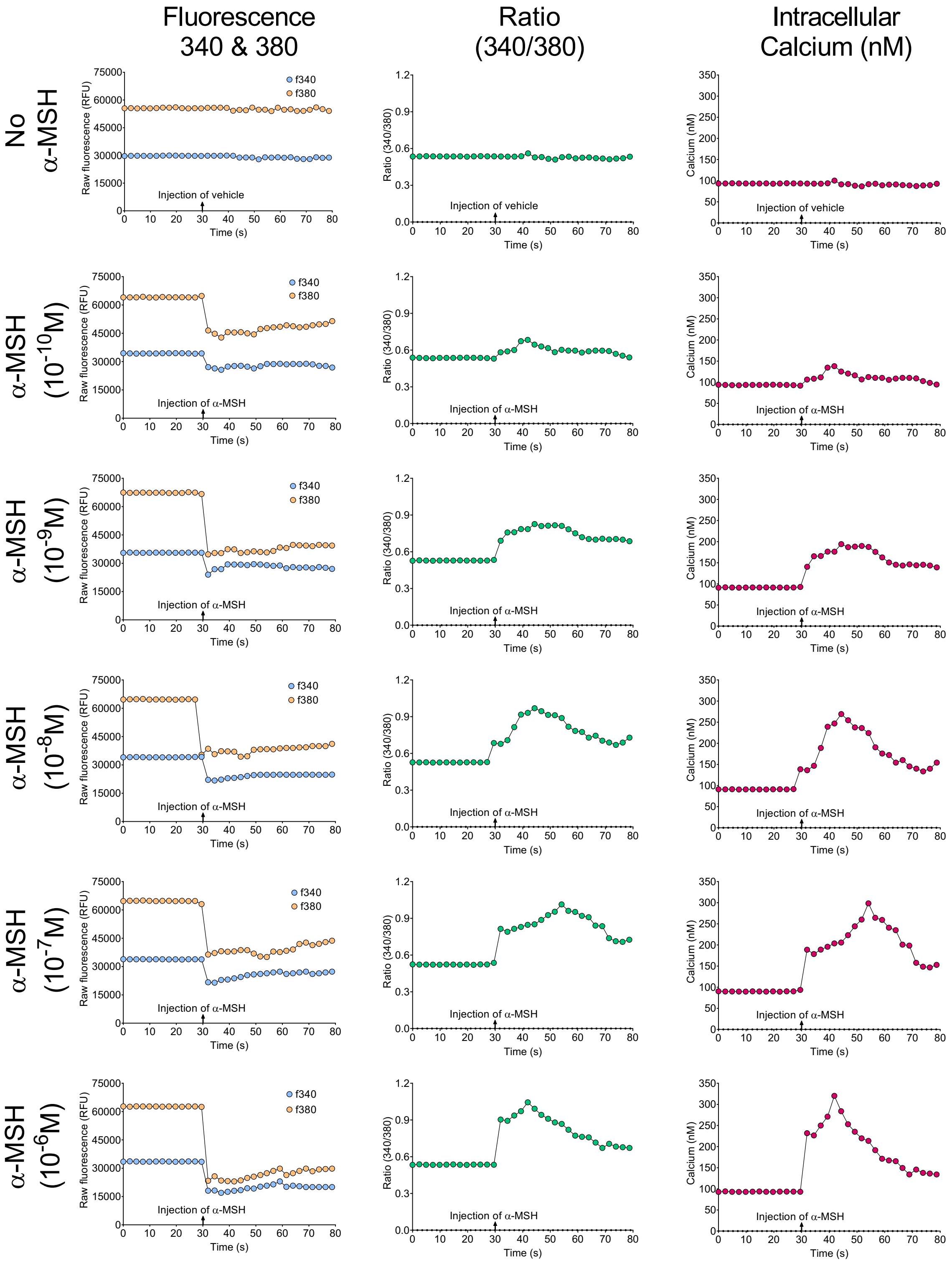

Supplementary Figure 3. Example of Fura-2 dynamic fluorescence changes induced by α-MSH for WT hMC4R transfected in HEK293 cells. Baseline fluorescence (left hand panel) for Fura-2 without Ca^{2+} bound (f380) and with Ca^{2+} bound (f380) was measured for 30s prior to injection of vehicle (no α-MSH) or increasing concentrations of α-MSH as

shown on the far left of the panels. Fluorescence at both wavelengths was then measured for 50s post-injection. The f340/f380 ratio is shown in the middle panel and $[Ca^{2+}]i$ is shown as nM in the right hand panel. The data shown are for one of three independent experiments performed on WT hMC4R stably transfected in HEK293 cells. A sigmoidal concentration response curve was generated by pooling the data from three independent experiments and is shown in **Figure 4B,C,E,F.**
