# Supplementary material for: A unique melanocortin-4-receptor signaling profile for obesity-associated constitutively active variants
Source: J Mol Endocrinol. 2023 Jun 12;71(1):e230008. doi: 10.1530/JME-23-0008 (PMC10304906; doi:10.1530/JME-23-0008)
Supplement: Supplementary Figure 4 [file supplementary_figure_4.pdf]

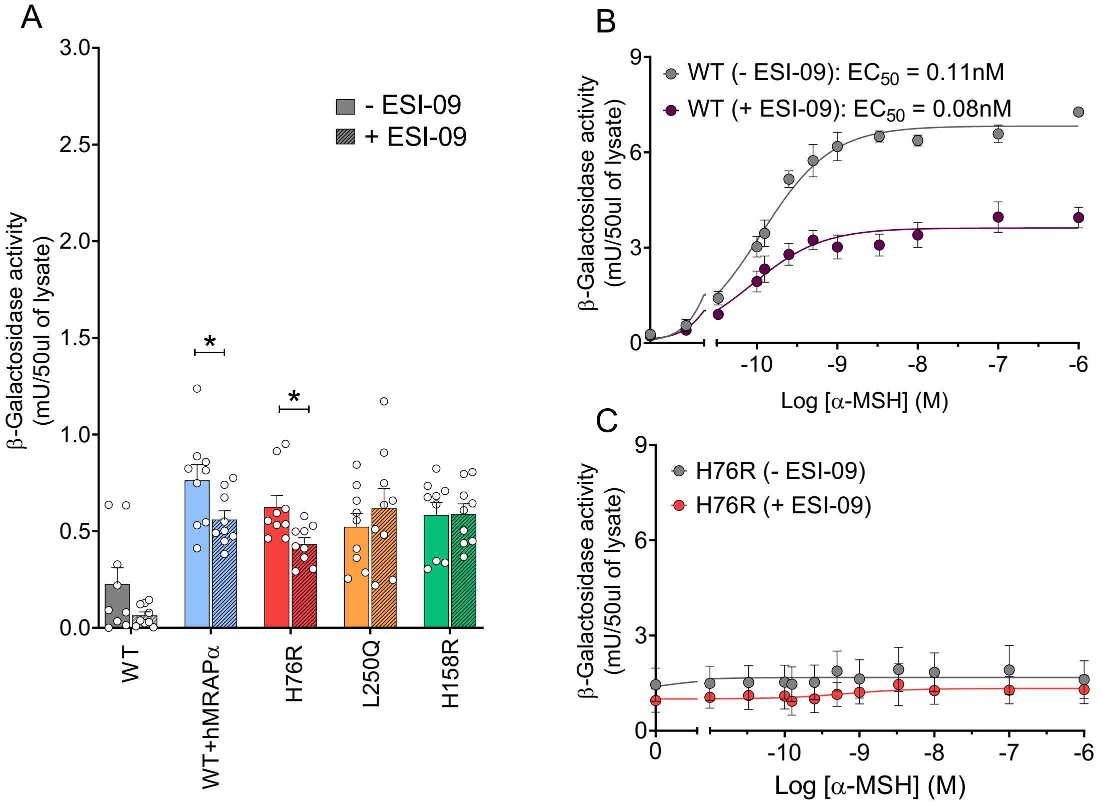

D

Variant	Baseline -/+ ESI-09 P value	Maximum -/+ ESI-09 P value	Log EC_{50} -/+ ESI-09 P value
WT	0.8692	<0.0001	0.4294
H76R	<0.0001		

Supplementary Figure 4. ESI-09 pre-treatment significantly reduced basal and maximum α-MSH induced CRE-β-Gal reporter gene activity for WT hMC4R, constitutive CRE-β-Gal reporter activity for H76R and constitutive activity for WT hMC4R co-expressed with hMRAPα. The CRE-β-Gal reporter assays were performed with triple transfections using DNAs for CRE-β-Gal reporter gene, WT hMC4R + pcDNA3.1, WT hMC4R + hMRAPα, + pcDNA3.1, H76R, L250Q + pcDNA3.1, and H158R + pcDNA3.1. We measured basal CRE-β-Gal reporter gene activity following pre-treatment of cells with vehicle or ESI-09 (**A**). Data from three independent experiments and shown as mean ± SEM. We performed pairwise student t-test between vehicle and ESI-09 pre-treated to determine significant differences for basal activity. *, $p < 0.05$; ****, $p < 0.0001$. We measured α-MSH induced CRE-β-Gal reporter gene activity for WT hMC4R (**B**) and H76R (**C**) following pre-treatment with vehicle or ESI-09. α-MSH concentration-response curves were fitted to raw

data for CRE-β-Gal reporter gene activity per well using GraphPad Prism. Summary of significance for comparative CRE-β-Gal reporter gene activity best-fit curve fitting for vehicle versus ESI-09 (**D**). We pooled data from three independent experiments and data are shown as mean ± SEM. The non-parametric sum of squares f-test was used to determine significance for parameters derived from curve fitting. The statistical significance for the maximum constitutive response for H76R (**B**) and WT hMC4R co-expressed with hMRAPα (**C**) was determined by averaging CRE-β-Gal reporter gene activity across all α-MSH concentrations for pre-treatment with vehicle or ESI-09 and tested the difference between vehicle and ESI-09 responses for significance using pairwise Student t test (**D**).
