# Supplementary material for: A unique melanocortin-4-receptor signaling profile for obesity-associated constitutively active variants
Source: J Mol Endocrinol. 2023 Jun 12;71(1):e230008. doi: 10.1530/JME-23-0008 (PMC10304906; doi:10.1530/JME-23-0008)
Supplement: Supplementary Table 1: Naturally occurring hMC4R variants selected for this study showing previously identified characteristics for each variant. BMI, body mass index; AC, adenylyl cyclase; RIA, radioimmunoassay. [file supplementary_table_1.pdf]

Supplementary Table 1: Naturally occurring hMC4R variants selected for this study showing previously identified characteristics for each variant. BMI, body mass index; AC, adenylyl cyclase; RIA, radioimmunoassay.

Category	hMC4R Variant	Gender	Age (Yrs)	BMI	Cell surface MC4R (% WT)	cAMP assay	Basal signal (% WT)	Agonist response (% WT)	Key references
Constitutively active: Obesogenic	H76R	F	-	~41	55/WT-like	RIA/CRE-Reporter	1700/~187	158/~135/~85	(Gillyard, et al. 2019; Moore, et al. 2014; Stutzmann, et al. 2008; Tao, et al. 2010; Wang and Tao 2011)
	S127L	M	14	37	WT-like/~50	RIA/AC/CRE Reporter/ AlphaScreen ™	WT-like/~250	<<50/57	(Fan and Tao 2009; Hinney, et al. 2003; Lubrano-Berthelier, et al. 2003; Paisdzior, et al. 2020; Valli-Jaakola, et al. 2004; Xiang, et al. 2006)
	P230L	-	-	33	~80	AC/RIA/CRE-Reporter	200-300	2700	(Fan and Tao 2009; Hinney et al. 2003; Xiang et al. 2006)
	L250Q	F	25	59	50-95	RIA/CRE-Reporter	156-1000	79	(Lubrano-Berthelier, et al. 2006; Moore et al. 2014; Proneth, et al. 2006; Stutzmann et al.

									2008; Tao et al. 2010; Vaisse, et al. 2000; Xiang et al. 2006)
	F280L	F	12	36	50	RIA/CRE-Reporter	~300	174	(Beckers, et al. 2010; Wang and Tao 2011)
	S295P	F	35	44	WT-like	CRE-Reporter	124	WT-like	(Lubrano-Berthelier et al. 2006; Moore et al. 2014; Stutzmann et al. 2008; Xiang, et al. 2010)
Non-constitutively active: Obesogenic	R7H	-	-	30	WT-like	CRE-Reporter	50	WT-like	(Srinivasan, et al. 2004; Xiang et al. 2010)
	R18L	-	-	30	95	CRE-Reporter	~40	WT-like	(Srinivasan et al. 2004; Xiang et al. 2010)
	D90N	F	11	32	WT-like/~60	CRE-reporter	WT-like	0/<<50	(Biebermann, et al. 2003; Buch, et al. 2009; Moore et al. 2014; Xiang et al. 2010)
	T150I	F	51	49	95/WT-like	CRE-Reporter	≤10/<50	56	(Lubrano-Berthelier et al. 2006; Vaisse et al. 2000; Xiang et al. 2006)
		M	52	50					

	A154D	F	43	43	95-115	CRE-Reporter	11	90	(Lubrano-Berthelier et al. 2006; Stutzmann et al. 2008; Xiang et al. 2010)
	R305S	-	-	34	~90	RIA/CRE-Reporter	40/WT-like	180/~39/WT-like	(Gillyard et al. 2019; Stutzmann et al. 2008; Wang and Tao 2011)
Constitutively active: Non-obesogenic	D146N	-	-	19	~20	RIA	~250/1400	~50/WT-like	(Stutzmann et al. 2008; Tao et al. 2010; Wang and Tao 2011)
	H158R	M	45	28	WT-like	AC/AlphaScreen™	532	142/147	(Hinney, et al. 2006; Paisdzior et al. 2020)
Protection from obesity	V103I	-	-	-	WT-like	AC	WT-like	WT-like	(Geller, et al. 2004; Hinney et al. 2003; Moore et al. 2014; Paisdzior et al. 2020; Vaisse et al. 2000)
	I251L	-	-	-	35-95	AC/CRE-Reporter	WT-like	WT-like	(Hinney et al. 2003; Hinney, et al. 1999; Moore et al. 2014; Vaisse et al. 2000; Xiang et al. 2006)

Beckers S, Zegers D, de Freitas F, Peeters AV, Verhulst SL, Massa G, Van Gaal LF, Timmermans JP, Desager KN & Van Hul W 2010 Identification and functional characterization of novel mutations in the melanocortin-4 receptor. *Obesity Facts* **3** 304-311.

Biebermann H, Krude H, Elsner A, Chubanov V, Gudermann T & Gruters A 2003 Autosomal-dominant mode of inheritance of a melanocortin-4 receptor mutation in a patient with severe early-onset obesity is due to a dominant-negative effect caused by receptor dimerization. *Diabetes* **52** 2984-2988.

Buch TR, Heling D, Damm E, Gudermann T & Breit A 2009 Pertussis toxin-sensitive signaling of melanocortin-4 receptors in hypothalamic GT1-7 cells defines agouti-related protein as a biased agonist. *Journal of Biological Chemistry* **284** 26411-26420.

Fan ZC & Tao YX 2009 Functional characterization and pharmacological rescue of melanocortin-4 receptor mutations identified from obese patients. *Journal of Cellular and Molecular Medicine* **13** 3268-3282.

Geller F, Reichwald K, Dempfle A, Illig T, Vollmert C, Herpertz S, Siffert W, Platzer M, Hess C, Gudermann T, et al. 2004 Melanocortin-4 receptor gene variant I103 is negatively associated with obesity. *American Journal of Human Genetics* **74** 572-581.

Gillyard T, Fowler K, Williams SY & Cone RD 2019 Obesity-associated mutant melanocortin-4 receptors with normal Galphas coupling frequently exhibit other discoverable pharmacological and biochemical defects. *Journal of Neuroendocrinology* **31** e12795.

Hinney A, Hohmann S, Geller F, Vogel C, Hess C, Wermter AK, Brokamp B, Goldschmidt H, Siegfried W, Remschmidt H, et al. 2003 Melanocortin-4 receptor gene: case-control study and transmission disequilibrium test confirm that functionally relevant mutations are compatible with a major gene effect for extreme obesity. *Journal of Clinical Endocrinology and Metabolism* **88** 4258-4267.

Hinney A, Schmidt A, Nottebom K, Heibult O, Becker I, Ziegler A, Gerber G, Sina M, Gorg T, Mayer H, et al. 1999 Several mutations in the melanocortin-4 receptor gene including a nonsense and a frameshift mutation associated with dominantly inherited obesity in humans. *Journal of Clinical Endocrinology and Metabolism* **84** 1483-1486.

Hinney A, Bettecken T, Tarnow P, Brumm H, Reichwald K, Lichtner P, Scherag A, Nguyen TT, Schlumberger P, Rief W, et al. 2006 Prevalence, spectrum, and functional characterization of melanocortin-4 receptor gene mutations in a representative population-based sample and obese adults from Germany. *Journal of Clinical Endocrinology and Metabolism* **91** 1761-1769.

Lubrano-Berthelier C, Durand E, Dubern B, Shapiro A, Dazin P, Weill J, Ferron C, Froguel P & Vaisse C 2003 Intracellular retention is a common characteristic of childhood obesity-associated MC4R mutations. *Human Molecular Genetics* **12** 145-153.

Lubrano-Berthelier C, Dubern B, Lacorte JM, Picard F, Shapiro A, Zhang S, Bertrais S, Hercberg S, Basdevant A, Clement K, et al. 2006 Melanocortin 4 receptor mutations in a large cohort of severely obese adults: prevalence, functional classification, genotype-phenotype relationship, and lack of association with binge eating. *Journal of Clinical Endocrinology and Metabolism* **91** 1811-1818.

Moore BS, Mirshahi UL, Yost EA, Stepanchick AN, Bedrin MD, Styer AM, Jackson KK, Still CD, Breitwieser GE, Gerhard GS, et al. 2014 Long-term weight-loss in gastric bypass patients carrying melanocortin 4 receptor variants. *PLoS ONE* **9** e93629.

Paisdzior S, Dimitriou IM, Schope PC, Annibale P, Scheerer P, Krude H, Lohse MJ, Biebermann H & Kuhnen P 2020 Differential Signaling Profiles of MC4R Mutations with Three Different Ligands. *International Journal of Molecular Sciences* **21** 1224.

Proneth B, Xiang Z, Pogozheva ID, Litherland SA, Gorbatyuk OS, Shaw AM, Millard WJ, Mosberg HI & Haskell-Luevano C 2006 Molecular mechanism of the constitutive activation of the L250Q human melanocortin-4 receptor polymorphism. *Chemical Biology and Drug Design* **67** 215-229.

Srinivasan S, Lubrano-Berthelier C, Govaerts C, Picard F, Santiago P, Conklin BR & Vaisse C 2004 Constitutive activity of the melanocortin-4 receptor is maintained by its N-terminal domain and plays a role in energy homeostasis in humans. *Journal of Clinical Investigation* **114** 1158-1164.

Stutzmann F, Tan K, Vatin V, Dina C, Jouret B, Tichet J, Balkau B, Potoczna N, Horber F, O'Rahilly S, et al. 2008 Prevalence of melanocortin-4 receptor deficiency in Europeans and their age-dependent penetrance in multigenerational pedigrees. *Diabetes* **57** 2511-2518.

Tao YX, Huang H, Wang ZQ, Yang F, Williams JN & Nikiforovich GV 2010 Constitutive activity of neural melanocortin receptors. *Methods in Enzymology* **484** 267-279.

Vaisse C, Clement K, Durand E, Hercberg S, Guy-Grand B & Froguel P 2000 Melanocortin-4 receptor mutations are a frequent and heterogeneous cause of morbid obesity. *Journal of Clinical Investigation* **106** 253-262.

Valli-Jaakola K, Lipsanen-Nyman M, Oksanen L, Hollenberg AN, Kontula K, Bjorbaek C & Schalin-Jantti C 2004 Identification and characterization of melanocortin-4 receptor gene mutations in morbidly obese finnish children and adults. *Journal of Clinical Endocrinology and Metabolism* **89** 940-945.

Wang ZQ & Tao YX 2011 Functional studies on twenty novel naturally occurring melanocortin-4 receptor mutations. *Biochimica et Biophysica Acta* **1812** 1190-1199.

Xiang Z, Proneth B, Dirain ML, Litherland SA & Haskell-Luevano C 2010 Pharmacological characterization of 30 human melanocortin-4 receptor polymorphisms with the endogenous proopiomelanocortin-derived agonists, synthetic agonists, and the endogenous agouti-related protein antagonist. *Biochemistry* **49** 4583-4600.

Xiang Z, Litherland SA, Sorensen NB, Proneth B, Wood MS, Shaw AM, Millard WJ & Haskell-Luevano C 2006 Pharmacological characterization of 40 human melanocortin-4 receptor polymorphisms with the endogenous proopiomelanocortin-derived agonists and the agouti-related protein (AGRP) antagonist. *Biochemistry* **45** 7277-7288.
