# Supplementary material for: A unique melanocortin-4-receptor signaling profile for obesity-associated constitutively active variants
Source: J Mol Endocrinol. 2023 Jun 12;71(1):e230008. doi: 10.1530/JME-23-0008 (PMC10304906; doi:10.1530/JME-23-0008)
Supplement: Supplementary Table 2: List of primers generated for SOE cloning of HA-hMC4R variants. [file supplementary_table_2.pdf]

Supplementary Table 2: List of primers generated for SOE cloning of HA-hMC4R variants.

Primer	Purpose	Nucleotide sequence
**HA-hMC4RForR1**	Binding pcDNA3.1 multiple cloning site upstream of the HA-epitope (Near NheI)	5’ gctagcgtttaaacttaagc
**HA-hMC4RRevR2**	Binding pcDNA3.1 multiple cloning site downstream of HA-epitope (Near XhoI)	5’ ggccctctagactcgagtta
**HA-hMC4RForR3**	Amplification of full length mutant HA-hMC4R (Near Asp7181 and KpnI)	5’ cttggtaccaccatgtaccc
**HA-hMC4RRevR3**	Amplification of full length mutant HA-hMC4R (Near 3’ HA-hMC4R and XhoI)	5’ gactcgagttaatatctgc
**HA-hMC4RR7HForR2**	Introducing CGT codon change to CAT (His)	5’ aactccacccaccatgggatgcacacttc
**HA-hMC4RR7HRevR1**		5’ gaagtgtgcatcccatggtgggtggagtt
**HA-hMC4RR18LForR2**	Introducing CGC codon change to CTC (Leu)	5’ gcacctctggaacctcagcagttacagac
**HA-hMC4RR18LRevR1**		5’ gtctgtaactgctgaggttccagaggtgc
**HA-hMC4RH76RForR2**	Introducing CAT codon change to CGT (Arg)	5’ gaacaagaatctgcgttcacccatgtac
**HA-hMC4RH76RRevR1**		5’ gtacatgggtgaacgcagattcttgttc
**HA-hMC4RD90NForR2**	Introducing GAT codon change to AAT (Asn)	5’ cttggctgtggctaatatgctggtgagcg
**HA-hMC4RD90NRevR1**		5’ cgctcaccagcatattagccacagccaag
**HA-hMC4RV103IForR2**	Introducing GTC codon change to ATC (Ile)	5’ gaaaccattatcatcaccccta
**HA-hMC4RV103IRevR1**		5’ tagggtgatgataatggtttc
**HA-hMC4RD146NForR2**	Introducing GAC codon change to AAC (Asn)	5’ cctgctttcaattgcagtgaacaggtac
**HA-hMC4RD146NRevR1**		5’ gtacctgttcactgcaattgaaagcagg
**HA-hMC4RT150IForR2**	Introducing ACT codon change to ATT (Ile)	5’ gcagtggacaggtactttattatcttctatgctctc
**HA-hMC4RT150IRevR1**		5’ gagagcatagaagataataaagtacctgtccactgc
**HA-hMC4RA154DForR2**	Introducing GCT codon change to GAT (Asp)	5’ ctatcttctatgatctccagtacc
**HA-hMC4RA154DRevR1**		5’ ggtactggagatcatagaagatag
**HA-hMC4RH158RForR2**	Introducing CAT codon change to CGT (Arg)	5’ gctctccagtaccgtaacattatgacag
**HA-hMC4RH158RRevR1**		5’ ctgtcataatgttacggtactggagagc
**HA-hMC4RP230LForR2**	Introducing CCC codon change to CTC (Leu)	5’ aagaggattgctgtcctcctcggcactggtg
**HA-hMC4RP230LRevR1**		5’ caccagtgccgaggaggacagcaatcctctt
**HA-hMC4RL250QForR2**	Introducing CTG codon change to CAG (Gln)	5’ ccttgaccatccagattggcgtctttg
**HA-hMC4RL250QRevR1**		5’ caaagacgccaatctggatggtcaagg
**HA-hMC4RI251LForR2**	Introducing ATT codon change to CTG (Leu)	5’ accatcctgcttggcgtcttt
**HA-hMC4RI251LRevR1**		5’ aaagacgccaagcaggatggt
**HA-hMC4RF280LForR2**	Introducing TTC codon change to TTG (Leu)	5’ ccatattgtgtgtgcttgatgtctcac
**HA-hMC4RF280LRevR1**		5’ gtgagacatcaagcacacacaatatgg
**HA-hMC4RS295PForR2**	Introducing TCA codon change to CCA (Pro)	5’ gtgtaatccaatcatcgatcctctg
**HA-hMC4RS295PRevR1**		5’ cagaggatcgatgattggattacac
**HA-hMC4RR305SForR2**	Introducing CGG codon change to AGC (Ser)	5’ gatttatgcactcagcagtcaagaactgagg
**HA-hMC4RR305SRevR1**		5’ cctcagttcttgactgctgagtgcataaatc
**HA-hMC4RS127LForR2**	Introducing TCG codon change to TTG (Leu)	5’ atgtcattgacttggtgatctgtagctcc
**HA-hMC4RS127LRevR1**		5’ ggagctacagatcaccaagtcaatgacat
