# Supplementary material for: A unique melanocortin-4-receptor signaling profile for obesity-associated constitutively active variants
Source: J Mol Endocrinol. 2023 Jun 12;71(1):e230008. doi: 10.1530/JME-23-0008 (PMC10304906; doi:10.1530/JME-23-0008)
Supplement: Supplementary Table 3: Biochemical Reagents [file supplementary_table_3.pdf]

Supplementary Table 3: Biochemical Reagents

REAGENT or RESOURCE	SOURCE	IDENTIFIER
Antibodies		
Mouse monoclonal Anti-HA.11	Covance Research Products	Cat#MMS-101P<br>RRID:AB_291261
Sheep AffinPure anti-mouse IgG peroxidase-conjugated	Jackson Immuno Research Labs	Cat#515-035-003<br>RRID:AB_2340295
Donkey AffinPure anti-rabbit IgG HRP conjugated	Jackson Immuno Research Labs	Cat#711-035-152<br>RRID:AB_10015282
Goat AffinPure anti-rabbit IgG peroxidase conjugated	Jackson Immuno Research Labs	Cat#111-035-003<br>RRID:AB_2313567
Rabbit monoclonal anti-pERK½	Cell Signalling Technology	Cat#4370<br>RRID:AB_2315112
Rabbit monoclonal anti-ERK½	Cell Signalling Technology	Cat#4695<br>RRID:AB_390779
Bacterial and virus strains		
Maximum Efficiency™ DH5alpha Competent Cells	Thermo Fisher Scientific	18258012
Chemicals, peptides, and recombinant proteins		
Dulbecco's Modified Eagle Medium (DMEM)	Invitrogen	12100046
Newborn calf serum (NCS)	Invitrogen	16010159
Fetal calf serum (FCS)	Invitrogen	10091148
HEPES	Invitrogen	15630080
Trypsin.05% EDTA	Invitrogen	25300054
Versene	Invitrogen	15040066
Penicillin and streptomycin (P/S)	Invitrogen	15070063
Fugene 6 transfection reagent	Invitrogen	PME2691
Geneticin (G418)	Invitrogen	11811031
Poly-L-lysine (PLL)	Sigma-Aldrich	P1399
Isobutylmethylxanthine (IBMX)	Sigma-Aldrich	15879
Ortho-Nitrophenyl-β-galactoside (ONPG)	Sigma-Aldrich	N1127
n-Dodecyl-β-D-maltoside (DDM)	Sigma-Aldrich	D4641
Ethylene glycol-bis (□-aminoethyl ether)- N,N,N′,N′-tetraacetic acid (EGTA)	Sigma-Aldrich	E3889
Rp-Adenosine 3',5'-cyclic monophos	Sigma Aldrich	A165
Ionomycin calcium salt	Sigma Aldrich	10634
ESI-09	Sigma Aldrich	SML0814
Reporter lysis buffer (RLB)	Promega	PME3971
b-galactosidase (β-Gal) Enzyme Assay	Promega	PME2000
Fura-2/AM	Molecular Probes	F1221
Pluronic acid F-127	Molecular Probes	P3000MP
Probenecid	Molecular Probes	P36400
Dyngo4a	Selleck Chemicals	S7163
Hank's balanced salt solution (HBSS)	Gibco	14025092

BM Blue POD Substrate	Roche Applied Science	11484281 001
DpnI restriction enzyme	Roche Applied Science	10742970001
Complete mini protease inhibitors	Roche Applied Science	11697498001
Polyvinylidene difluoride (PVDF) membrane	Roche Applied Science	3010040001
PhosStop	Roche Applied Science	4906837001
Mini cell scrapers	LEAP Biosciences	NC0140511
β-mercaptoethanol (β-ME)	BioRad Laboratories	1610710
Precision Plus Unstained Standard	BioRad Laboratories	1610363
Mini-PROTEAN TGX Stain-free 10% and 15 well pack	BioRad Laboratories	4568036
Mini-PROTEAN TGX Stain-free 10% and 10 well pack	BioRad Laboratories	4568033
Clarity™ Western ECL substrate	BioRad Laboratories	1705061
Bio-Rad DC protein assay kit	BioRad Laboratories	5000116
2 X Laemnli Sample Buffer	BioRad Laboratories	1610737
iProof High Fidelity DNA Polymerase	BioRad Laboratories	1725301
Poly-Prep(R) Chromatography columns, Pkg of 50	BioRad Laboratories	73115500
AG(R) 50W-8 Cation Exchange Resin	BioRad Laboratories	1435441
α-MSH	Bachem Holding AG	H1075
Costar® 96-well black clear-bottom Tissue Culture treated plate	Thermo Fisher Scientific	COR3603
Costar® 24-well clear Tissue Culture treated plate	Thermo Fisher Scientific	353047
Nunc 96-well protein assay plate	Thermo Fisher Scientific	269620
Methanol Pronalys AR ACS	Thermo Fisher Scientific	BSPML868
Adenine [2,8-3H]	SciMed Limited	NET06300
Optiphase supermix cocktail (scintillation fluid)	SciMed Limited	1200439
Aluminium Oxide activated Neutral	Global Sciences	SCARAL08351000
Critical commercial assays		
PureLink™ HiPure Plasmid Maxiprep Kit	Thermo Fisher Scientific	K210007
Experimental models: Cell lines		
HEK293 cell line	ATCC	CRL-1573
GT1-7 mouse hypothalamic cell line	Gift from Dr. Pamela Mellon	N/A
Oligonucleotides		
For PCR primers used to construct hMC4R variants see **Table S2**.	This study	N/A
Recombinant DNA		
HA-hMC4R-WT Plasmid	Missouri S&T cDNA Resource Center	MCR040TN00
CRE-β-Gal reporter gene Plasmid	Gift from Dr. Roger Cone [1]	N/A

pcDNA3.1	Thermo Fisher Scientific	V79020
HA-hMC4R R7H	This study	N/A
HA-hMC4R R18L	This study	N/A
HA-hMC4R H76R	This study	N/A
HA-hMC4R D90N	This study	N/A
HA-hMC4R V103I	This study	N/A
HA-hMC4R D146N	This study	N/A
HA-hMC4R T150I	This study	N/A
HA-hMC4R I154D	This study	N/A
HA-hMC4R H158R	This study	N/A
HA-hMC4R P230L	This study	N/A
HA-hMC4R L250Q	This study	N/A
HA-hMC4R I251L	This study	N/A
HA-hMC4R F280L	This study	N/A
HA-hMC4R S295P	This study	N/A
HA-hMC4R R305S	This study	N/A
HA-hMC4R S127L	This study	N/A
hMRAPa	[2]	N/A
hMRAP2	[2]	N/A
Software and algorithms		
GraphPad Prism 7.0	Scientific Software	N/A
ImageLab	BioRad Laboratories	N/A
MARS Data Analysis Version 3.32	BMG LABTECH	N/A
