# Supplementary material for: A unique melanocortin-4-receptor signaling profile for obesity-associated constitutively active variants
Source: J Mol Endocrinol. 2023 Jun 12;71(1):e230008. doi: 10.1530/JME-23-0008 (PMC10304906; doi:10.1530/JME-23-0008)
Supplement: Supplementary Table 4 [file supplementary_table_4.pdf]

Supplementary Table 4. Summary of CRE-β-Gal reporter gene results for Basal signaling and α-MSH concentration-dependent signaling for WT hMC4R co-expressed with either hMRAPα or hMRAP2 and hMC4R variants, compared to WT hMC4R transfected in HEK293 cells.

**hMC4R variant or co-expression**	**Basal signaling assay**	**α-MSH concentration-dependent signaling assay**		
		**Baseline Response**	**Log EC_{50}**	**Maximum Response**
WT + hMRAP2	→	→	→	↓
WT + hMRAPα	↑	↑	Nil Response	
H76R	↑	↑	Nil Response	
L250Q	↑	↑	Nil Response	
H158R	↑	↑	→	↓
T150I	→	→	↑	↓
A154D	→	→	↑	↓
R305S	→	→	↑	↓
R18L	→	→	→	→
R7H	→	→	→	↑
I251L	→	→	→	↓
V103I	→	→	→	↓

(→); Not significantly different from WT hMC4R

(↑); Significantly increased compared with WT hMC4R

(↓); Significantly decreased compared with WT hMC4R

Constitutive activity = ↑ Basal or ↑ Baseline response

Impaired α-MSH signaling = Nil Response

Partial impaired α-MSH signaling = ↑ EC50 and/or ↓ Maximum Response
