# Supplementary material for: A unique melanocortin-4-receptor signaling profile for obesity-associated constitutively active variants
Source: J Mol Endocrinol. 2023 Jun 12;71(1):e230008. doi: 10.1530/JME-23-0008 (PMC10304906; doi:10.1530/JME-23-0008)
Supplement: Supplementary Table 6 [file supplementary_table_6.pdf]

Supplementary Table 6. Summary of $[Ca^{2+}]i$ results for Basal signaling and α-MSH concentration-dependent signaling for WT hMC4R co-expressed with hMRAPα and hMC4R variants, compared to WT hMC4R transfected in HEK293 cells.

hMC4R variant or co-expression	Basal signaling assay	α-MSH concentration-dependent signaling assay		
		**Baseline Response**	**Log EC_{50}**	**Maximum Response**
WT + hMRAPα	↑	→	↓	→
H76R	↑	↑	↓	→
L250Q	↑	↑	↓	→
H158R	↑	→	↓	→
R18L	→	→	→	→
R7H	→	→	→	→
I251L	↓	→	→	↑
V103I	↓	→	→	↑

(→); Not significantly different from WT hMC4R

(↑); Significantly increased compared with WT hMC4R

(↓); Significantly decreased compared with WT hMC4R

Constitutive activity = ↑ Basal or ↑ Baseline response

Partial impaired α-MSH signaling = ↑ EC50 and/or ↓ Maximum Response
