# Supplementary material for: A unique melanocortin-4-receptor signaling profile for obesity-associated constitutively active variants
Source: J Mol Endocrinol. 2023 Jun 12;71(1):e230008. doi: 10.1530/JME-23-0008 (PMC10304906; doi:10.1530/JME-23-0008)
Supplement: Supplementary Table 7 [file supplementary_table_7.pdf]

Supplementary Table 7. Percentage change in cell surface and total cellular hMC4R protein expression for WT hMC4R co-expressed with either hMRAPα or hMRAP2 and hMC4R variants, compared to WT hMC4R.

hMC4R variant or co-expression	Cell surface protein expression compared to WT hMC4R (%)		Total protein expression compared to WT hMC4R (%)	
WT+hMRAPα	↓	64.9****	↓	65.8**
WT+hMRAP2	↓	29.7**	↑	24.2
R7H	↓	39.8****	↓	67.5*
R18L	↓	50.3****	↓	34.4
H76R	↓	43.8****	↓	54.9***
D90N	↓	46.5****	↓	71.1***
V103I	↑	15.8	↓	9.58
S127L	↓	40.1****		NM
D146N	↓	84.8****		NM
T150I	↓	44.4****	↓	67.8***
A154D	↓	39.3****	↓	66.6***
H158R	↑	27.1**	↓	8.06
P230L	↓	64.9****		NM
L250Q	↓	63.6****	↓	74.2***
I251L	↓	9.61	↓	17.2
F280L	↓	89.2****		NM
S295P	↓	29.1**		NM
R305S	↓	57.6****	↓	74.7***

NM = Not measured.
Significant difference from WT hMC4R;
*, $p < 0.05$; **, $p < 0.01$; ***, $p < 0.001$; ****, $p < 0.00001$
